# Proteomic analysis reveals a role of melatonin in promoting cucumber seed germination under high salinity by regulating energy production

**DOI:** 10.1038/s41598-017-00566-1

**Published:** 2017-03-29

**Authors:** Na Zhang, Hai-Jun Zhang, Qian-Qian Sun, Yun-Yun Cao, Xingsheng Li, Bing Zhao, Ping Wu, Yang-Dong Guo

**Affiliations:** 1 College of Horticulture, China Agricultural University, Beijing, China; 2 Beijing Vegetable Research Center, Beijing Academy of Agriculture and Forestry Sciences, National Engineering Research Center for Vegetables, Beijing, China; 3 Shandong Provincial Key Laboratory of Cucurbitaceae Vegetable Biological Breeding, Shandong Huasheng Agriculture Co. Ltd, Shandong, China

## Abstract

Seed germination is a critical and complex process in the plant life cycle. Although previous studies have found that melatonin can promote seed germination under salt stress, the involvement of melatonin in the regulation of proteomic changes remains poorly understood. In this study, a total of 157 proteins were significantly influenced (ratio ≥ 2 or ≤ −2) by melatonin during seed germination under salt stress using a label-free quantitative technique. Our GO analysis revealed that several pathways were obviously regulated by melatonin, including ribosome biosynthesis, lipid metabolism, carbohydrate metabolism, and storage protein degradation. Not only stress-tolerant proteins but also proteins that produce ATP as part of glycolysis, the citric acid cycle, and the glyoxylate cycle were upregulated by melatonin. Overall, this study provides new evidence that melatonin alleviates the inhibitory effects of NaCl stress on seed germination by promoting energy production. This study is the first to provide insights at the proteomic level into the molecular mechanism of melatonin in response to salt stress in cucumber seeds. This may be helpful to further understand the role of melatonin in cucumber seed germination under stress conditions.

## Introduction

Melatonin (N-acetyl-5-methoxytryptamine) is a naturally occurring compound that has been detected in the seeds, roots, fruits, and leaves of plants^1, 2^. Exogenous melatonin can alleviate biotic and abiotic stresses such as pathogen attack, extreme temperature, excess copper, intense light, salinity, drought, and senescence^3–11^. In recent years, increasing effort has been focused on the roles of melatonin in plants. Our previous work revealed a partial mechanism for melatonin in promoting seed germination by regulating plant hormone ABA and GA interactions under salt stress^8^, but information regarding melatonin in the regulation of seed germination is still lacking.

It is clear that seed germination is very important to the plant because it is a critical stage of regeneration that directly determines the establishment of the next-generation of plants. Seed germination is a complex and critical process in the life cycle of higher plants. By definition, seed germination commences with the uptake of water and is completed with the emergence of the radicle from the seed coat^12^. Seed germination is determined by both genetic and environmental factors^13^. In general, the progress of seed germination can be divided into three phases. In the first phase (phase I, fast water uptake), there is fast water uptake by the dry seed until all of the cell contents of seed are fully hydrated. The second phase (phase II, metabolism reactivation) is a period of limited water uptake, and seed germination is not completed. During the third phase (phase III, radicle emergence), the seed continues to absorb water until germination is completed^12^. Among the three phases, phase II is the most critical because all necessary metabolic pathways and physiological processes are reactivated and germination is initiated^14^. Seed germination involves many events, such as proteolysis, macromolecular synthesis, respiration, and cell elongation^12, 15^. However, the key events that regulate the completion of seed germination have yet to be determined^16^.

Proteomic analysis provides an important tool that can be used to investigate the functions of melatonin in plants. Proteomic analysis can help to identify specific proteins that are regulated by exogenous melatonin during specific biological responses or in certain processes. Melatonin was shown to regulate 309 proteins during leaf senescence in *Malus hupehensis*, and most of which exhibit hydrolase activity^17^. Melatonin was also shown to significantly influence levels of 76 proteins upon H_2_O_2_ treatment in Bermuda grass, and metabolic pathway analysis has shown that several pathways were markedly enhanced by melatonin treatment, including polyamine metabolism, major carbohydrate metabolism, photosynthesis, redox, and amino acid metabolism^18^. The metabolic pathway of seed germination is highly complex. Previous work has reported the activity of key enzymes in several critical processes during seed germination, such as the pentose phosphate pathway (PPP), glycolysis, the tricarboxylic acid cycle (TCA cycle), and amino acid metabolism^19^. Starch hydrolysis and sucrose transport have been proven to be important during wheat seed germination^20^. Several large-scale -omics methods (such as transcriptomics, proteomics, and metabolomics) have been applied to investigate the mechanisms of seed germination and have made great achievements^21^. However, most previous efforts have not focused on how melatonin affects proteomic changes. We previously investigated how exogenous melatonin influences seed germination under salt stress^8^. When compared with untreated seeds melatonin-pretreated seeds had a significantly higher germination rate in addition to higher activity of superoxide dismutase (SOD), catalase (CAT), and peroxidase (POD). We also investigated abscisic acid (ABA) and gibberellin acid (GA) biosynthesis and catabolism during seed germination as well as the expression levels of genes involved in ABA and GA biosynthesis and catabolism. Compared to NaCl treatment, melatonin induced rapid, significant decreases in ABA content and increases in GA (especially GA_4_) content during the early stage of germination. Data from previous studies have provided evidence that exogenous melatonin can alleviate the inhibitory effects of NaCl stress on germination. Based on this phenomenon, we further explored how exogenous melatonin might alter metabolism during seed germination under salt stress. Therefore, in this study, we used a label-free quantitative technique to identify and quantitate proteins involved in the cellular response to salt stress and melatonin. We attempted to find potential regulatory proteins and possible biological processes regulated by melatonin during seed germination. Together with our previous results, these proteomic data provide fundamental insight for future studies regarding the functions of melatonin in seed germination under adverse conditions.

## Results

### Melatonin promotes seed germination under high salinity

**Figure 1:**
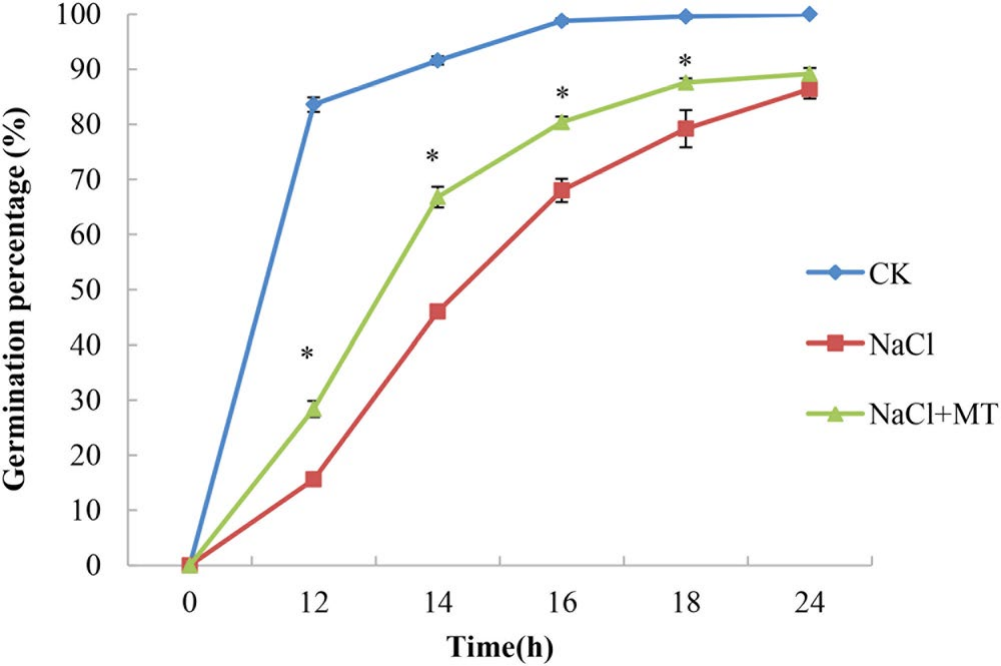
Melatonin promotes seed germination under high salinity. Melatonin (1 μM) promotes seed germination under NaCl stress. Vertical bars represent ± S.E (n = 3). *Significant difference between different treatments at P < 0.05 based on Duncan’s multiple range test.

We have previously investigated how exogenous melatonin influences seed germination in cucumber under salt stress^8^. The melatonin content significantly increased during the first 14 hr then decreased to a relative steady level under normal conditions^8^. Seeds primed in 1 μM melatonin for 24 hr had melatonin levels approximately nine fold higher than unprimed seeds. During germination, melatonin content decreased while alleviating the inhibitory effects of high salinity^8^. In this study, we carried out a similar germination test. Our data showed that the germination of cucumber seeds was significantly influenced by NaCl stress (*P* < 0.05, Fig. 1). Nearly, 83.6% of seeds germinated after 12 hr of incubation under normal conditions (CK), whereas only 15.6% of seeds under NaCl stress germinated. However, the percentage of germinated seeds was 28.4% for seeds pretreated with melatonin, 12.8% higher than seeds in NaCl treatment. NaCl stress decreased the seed germination percentage by 46% at 14 hr of incubation. Treatment with 1 μM melatonin (NaCl + MT) was the most effective at alleviating NaCl stress and produced a 22.8% increase in germination compared to NaCl treatment. These results indicate that melatonin has a positive role in alleviating NaCl stress during seed germination (Fig. 1). Based on this phenomenon, we used a label-free quantitation approach to seek more information about how exogenous melatonin application might alter metabolic processes in germinating cucumber seeds.

### Proteins Quantification and Analysis

**Figure 2:**
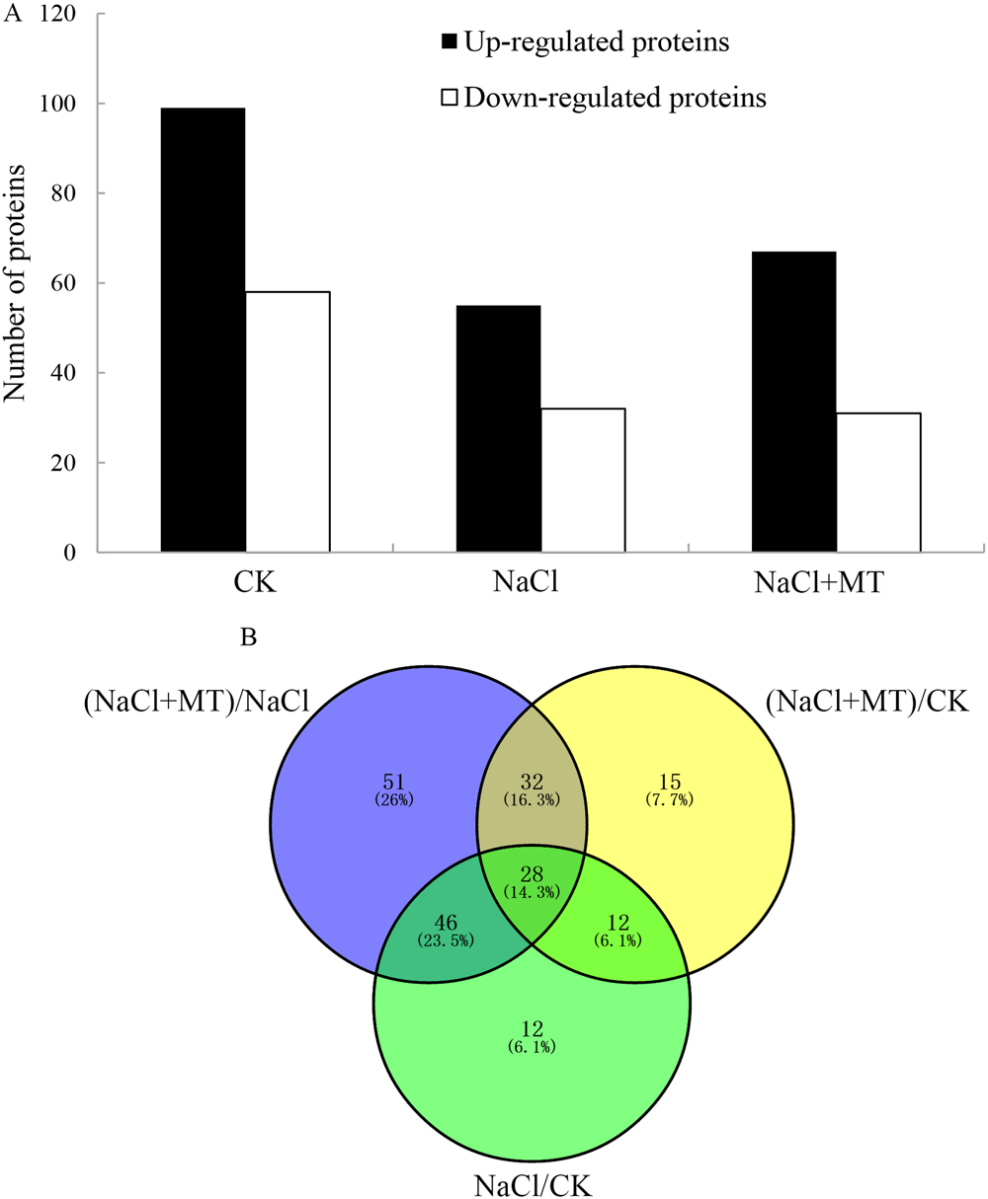
Distribution of differentially expressed proteins by melatonin and NaCl stress grass. (**A**) The number of differentially expressed proteins (fold change ≥  ± 2.0 and P value < 0.05). (**B**) Venn diagram showing the number of overlapping proteins that were differentially expressed between melatonin-treated seeds and NaCl stress conditions.

To investigate the potential mechanism of melatonin in promoting cucumber seed germination under salt stress, dynamic profiling of total protein was employed to identify proteins affected by melatonin treatment and salt stress. Proteomic analysis was performed using a label-free system after 14 hr of incubation, and a total of 472 proteins were detected. We compared the total protein extracted from seeds that had undergone NaCl stress treatment and exogenous melatonin treatment to that of control. The proteins were considered to be identified and useful when they were detected in any two or three treatments. All of the relative quantified proteins were divided into two categories: proteins with quantitative ratio over 2 were considered to be up-regulated (*P* < 0.05), while proteins with quantitative ratios <0.5 were considered to be down-regulated (*P* < 0.05). The numbers of significantly expressed proteins are summarized in Table S1 and Fig. 2. In total, 157 proteins were significantly regulated by melatonin under salt stress (Fig. 2A). Venn diagram analysis showed that the levels of 28 proteins were influenced by melatonin under both control and NaCl stress conditions (Fig. 2B). According to GO analysis (see Supplementary File 1), these proteins were classified by biological process, molecular function, and cellular component based on sequence identity (Fig. S1). The ‘biological processes’ represented ranged from metabolism to development (Fig. S1A), while ‘molecular functions’ covered activities from catalysis to transcription factors (Fig. S1B) and ‘cellular components’ included various cell parts and specific organelles (Fig. S1C). The assigned classifications were helpful in understanding the functions of melatonin in regulating seed germination under salt stress.

### Melatonin up-regulates the proteins associated with seed imbibition

To further study the function of proteins affected by melatonin, several proteins significantly regulated by melatonin or NaCl stress are annotated in Table 1. A large amount of energy and carbon skeletons are required during seed germination^16, 22^, and energy production is an early event during seed germination^23^. Seeds contain three types of stored reserves, including storage protein, lipid and starch^23^. Storage proteins are important nutrient contents in seeds. During germination, the degradation of storage proteins provides a source of sustenance and energy for seedling growth later^23^. Putative globulin or globulin-like protein^24, 25^ and globulin 2^26^ were found to be differentially regulated during seed germination. Levels of globulin precursor, cupin family protein, globulin and vicilin-like embryo storage protein, which come from breakdown of storage proteins, were increased in rice seeds under high temperature and ABA conditions^23^. In this study, five storage proteins were identified. NaCl stress down-regulated the degradation of storage proteins (P13744, Q8W1C2, Q9ZWI3, Q39651, F2YML9) by 21.82-, 13.65-, 7.69-, 21.46-, and 8.47-fold, respectively (Table 1). After treatment with exogenous melatonin, we found that two globulin-like proteins (P13744 and Q8W1C2) and two vicilin-like proteins (Q9ZWI3 and Q39651) were significantly down-regulated by 20.0-, 20.0-, 6.7-, and 3.45- fold, respectively (Table 1). These data showed that, melatonin promoted the degradation of storage proteins to produce energy for seed germination. This result was similar to a previous observation in seed germination^23–26^.

**Table 1:** Proteins involved in the biological process after melatonin treatment under salt stress.

Description	Protein ID	(NaCl + MT)/NaCl		NaCl/CK	
		Value	*P*	Value	*P*
***Response to stress***					
14-3-3 protein	Q5UFR1	24.64↑	0.00000	—	—
14-3-3-like protein gf14 iota	W9S288	31.92↑	0.00004	—	—
14-3-3-like protein gf14 omega	Q39558	0.66	0.22418	—	—
26 s proteasome non-ATPase regulatory subunit 4 homolog	M5W204	2.10↑	0.00011	0.42↓	0.00427
26 s proteasome regulatory subunit rpn13-like isoform x1	I1K657	3.724↑	0.00531	—	—
Ubiquitin domain-containing protein dsk2a-like	A0A067L974	0.13↓	0.00006	0.42↓	0.11805
Ubiquitin domain-containing protein dsk2a-like	B9H660	0.34↓	0.00007	0.38↓	0.00339
Polyubiquitin-a isoform x2	A0A072UMK2	0.15↓	0.00000	1.83	0.14659
Ubiquitin-protein E3 ligase	B3U2B1	3.10↑	0.00306	0.41↓	0.01479
Small ubiquitin-related modifier 1	M5WJN3	0.17↓	0.01948	0.26↓	0.23354
Small ubiquitin-related modifier 1-like	O23759	0.32↓	0.00035	—	—
28 kda heat- and acid-stable phosphoprotein	G7IFI0	0.19↓	0.00201	0.59	0.13080
Heat shock 70 kda protein	G7L007	10.95↑	0.00003	—	—
Heat shock 70 kda protein	M5W6U5	2.22↑	0.00015	3.24↑	0.00004
Heat shock 70 kda protein 17-like	B9HDE5	2.72↑	0.00006	—	—
Heat shock cognate protein 80	W9RXY8	3.86↑	0.00007	—	—
Heat shock protein 70	Q9M4E7	11.16↑	0.00619	10.99↑	0.03789
Heat shock protein 70 cognate	B9GVM4	—	—	2.15↑	0.00333
Heat shock protein 83	A0A067JRU1	5.01↑	0.00009	1.63	0.11292
Heat shock 70 kda mitochondrial	A0A067KUJ3	3.06↑	0.00016	—	—
Hsp70 nucleotide exchange factor fes1-like	A0A067KV00	0.16↓	0.00053	0.31↓	0.01468
Hsp70-hsp90 organizing protein 3	I1J9 × 8	0.25↓	0.04886	0.59	0.29238
Hsp70-hsp90 organizing protein 3-like	I1K0K7	0.18↓	0.00009	0.50	0.00470
Small heat shock chloroplastic-like	B9RMP5	2.23↑	0.00154	—	—
Small heat shock chloroplastic-like isoform x2	H6TB40	5.76↑	0.02131	2.84↑	0.07108
Kda class i heat shock protein	A0A072UYS2	5.34↑	0.00010	15.67↑	0.00119
Kda class i heat shock protein	P19243	16.89↑	0.00000	26.26↑	0.00005
Kda class ii heat shock	H6TB44	19.34↑	0.00772	—	—
Kda class iv heat shock protein	H6TB46	9.24↑	0.02695	—	—
Glutaredoxin-like protein	U3RGD2	0.48↓	0.00718	—	—
Glutathione peroxidase	B6DQ61	2.50↑	0.01323	—	—
Glutathione peroxidase mitochondrial	B9RCA6	0.80	0.00425	2.40↑	0.00035
Peroxidase 2-like	Q39650	3.08↑	0.01516	—	—
Peroxidase 2-like	Q6UBM4	2.94↑	0.00001	0.47↓	0.01591
Peroxiredoxin- chloroplastic	A9P8D8	5.04↑	0.00006	—	—
Peroxiredoxin family protein	B9HII6	2.17↑	0.00651	5.19↑	0.00138
Peroxiredoxin family protein	A0A067KWB6	1.62	0.07771	3.40↑	0.01039
Peroxygenase	B0F824	5.48↑	0.00047	39.23↑	0.00023
Sulfite oxidase	A0A072U725	8.09↑	0.00004	—	—
Superoxide dismutase	Q6QGY4	13.20↑	0.00012	—	—
***Cellular nitrogen compound biosynthetic process***					
40 s ribosomal protein s10-like	W9QS28	10.59↑	0.00008	—	—
40 s ribosomal protein s12-like isoform x2	A0A067L2F9	6.60↑	0.00005	—	—
40 s ribosomal protein s20–2	A9PAL8	4.85↑	0.00001	4.40↑	0.00023
40 s ribosomal protein s21–2	B9RFA5	9.81↑	0.00139	—	—
40 s ribosomal protein s5	O65731	43.20↑	0.00061	—	—
60 s ribosomal protein l5	Q6UNT2	0.32↓	0.00645	0.56	0.29084
60 s ribosomal protein l9	A0A067KWC5	6.20↑	0.00036	—	—
Nicotinamide mononucleotide adenylyltransferase	W9RZ99	5.30↑	0.00002	3.68↑	0.00001
Eukaryotic translation initiation factor 3 subunit j-like	M5WBU5	0.17↓	0.00059	0.47↓	0.03185
Elongation factor 1-alpha	V5IV18	9.25↑	0.00049	4.08↑	0.00098
***Storage proteins***					
Edestin 2	P13744	0.05↓	0.00001	21.82↑	0.00001
Legumin a	Q8W1C2	0.05↓	0.00000	13.65↑	0.00002
Vicilin gc72-a	Q9ZWI3	0.29↓	0.00000	7.69↑	0.00000
Vicilin-like antimicrobial peptides 2–2	Q39651	0.15↓	0.00005	21.46↑	0.00001
Vicilin-like protein	F2YML9	—	—	8.47↑	0.00038
***Cell part***					
Oleosin kda-like	B9GI54	0.43↓	0.00001	—	—
Oleosin kda-like	Q84T21	0.41↓	0.00008	2.72↑	0.01578
Tubulin beta-2 chain	B9S382	9.71↑	0.00012	0.60	0.00280
Actin-7	A0A067JQD9	19.51↑	0.00010	2.40↑	0.00833
***Carbohydrate metabolic process***					
Fructose-bisphosphate aldolase, cytoplasmic isozyme 1	A0A067KLE6	2.33↑	0.00046	3.20↑	0.00034
Fructose-bisphosphate aldolase, cytoplasmic isozyme-like	I1LZG1	2.38↑	0.01167	6.15↑	0.00003
Glyceraldehyde-3-phosphate dehydrogenase	E1B2J6	8.37↑	0.00003	—	—
Malate dehydrogenase mitochondrial	P17783	10.62↑	0.00000	7.74↑	0.00000
Cytosolic phosphoglycerate kinase family protein	B9HY30	10.79↑	0.02090	19.00↑	0.05771
Triosephosphate isomerase chloroplastic	A0A072U2W1	6.60↑	0.00174	—	—
Triosephosphate isomerase cytosolic	A0A067LKT3	7.05↑	0.00842	14.10↑	0.15009
Triosephosphate isomerase cytosolic	B9GJN0	8.23↑	0.00026	11.29↑	0.00307
Triosephosphate isomerase cytosolic	Q38IW8	10.64↑	0.00037	11.03↑	0.00048
Glucan endo- -beta-glucosidase 4	W9RG25	0.19↓	0.00031	0.29↓	0.04885
Lysosomal alpha-mannosidase isoform x1	A0A075CA98	4.21↑	0.00017	4.33↑	0.00378
Enolase	A0A067JHW3	6.84↑	0.00003	9.15↑	0.00003
Phosphoglycerate kinase	A1BQH1	11.23↑	0.00001	18.09↑	0.00003
Phosphoglycerate kinase cytosolic	I1MJC7	4.04↑	0.00160	10.82↑	0.00067
Glycosyl hydrolase family 17 family protein	B9H3B0	2.19↑	0.01513	0.30↓	0.02392
Alpha-xylosidase 1-like	W9SB70	6.36↑	0.00003	7.26↑	0.00008
Ribulose-phosphate 3- cytoplasmic isoform	M5WTG1	5.86↑	0.07207	1.89	0.34807
UTP–glucose-1-phosphate uridylyltransferase	Q19TV8	0.29↓	0.00084	2.90↑	0.07416
Isocitrate dehydrogenase	B9GHS2	2.03↑	0.01631	—	—
5-methyltetrahydropteroyltriglutamate–homocysteine methyltransferase	M5WFB9	12.42↑	0.00693	—	—
***Lipid metabolic process***					
Aspartic proteinase	O04057	4.09↑	0.00003	15.40↑	0.00009
Corticosteroid 11-beta-dehydrogenase	G7K984	6.95↑↑	0.00029	—	—
Glucose and ribitol dehydrogenase homolog 1	A0A067JL19	13.81↑	0.00002	13.81↑	0.00002
Triosephosphate isomerase chloroplastic	A0A072U2W1	6.60↑	0.00174	—	—
Triosephosphate isomerase cytosolic	A0A067LKT3	7.05↑	0.00842	14.10↑	0.15009
Triosephosphate isomerase cytosolic	B9GJN0	8.23↑	0.00026	11.29↑	0.00307
Triosephosphate isomerase cytosolic	Q38IW8	10.64↑	0.00037	11.03↑	0.00048

“↑” indicates increased proteins, and “↓”indicates significant decreased protein (fold change ≥ 2 or ≤ −2 and P value < 0.05). “−“indicates not detected.

### Melatonin regulates the cell elongation under salt stress

In *A. thaliana* seeds, tubulin 𝛼-2, 𝛼-3, 𝛼-4, 𝛼-5 were increased with GA_4+7_ treatment^27^ one day after imbibition. Salicylic acid (SA) markedly improved 𝛼-3, 𝛼-5 content during seed germination under salt stress^28^. Tubulin *β*-2 was also found to accumulate during seed priming^29^. Cortical microtubules are also formed in the radicle prior to germination in both tomato and cucumber^30^. Here, we found that exogenous melatonin significantly increased the abundance of hypothetical tubulin *β* chain (B9S382) and actin 7 (A0A067JQD9) under salt stress by approximately 9.7- and 19.5-fold, respectively, indicating a positive role for melatonin in cell elongation during seed germination (Table 1).

### Melatonin regulates the proteins associated with stress response

Plants respond to various stresses by producing heat shock proteins (HSPs) as an important adaptive mechanism^31^. HSPs act as molecular chaperones to help protect cells against stress by repairing stress-damaged proteins^32, 33^. HSP70 is a confirmed biomarker of NaCl stress^34^. Approximately 15 HSPs were observed in our study, and, among them, 11 were increased after melatonin treatment (including five HSP70 family members). The data in this study indicated that melatonin regulated HSPs to protect seed germination under salt stress.

**Figure 3:**
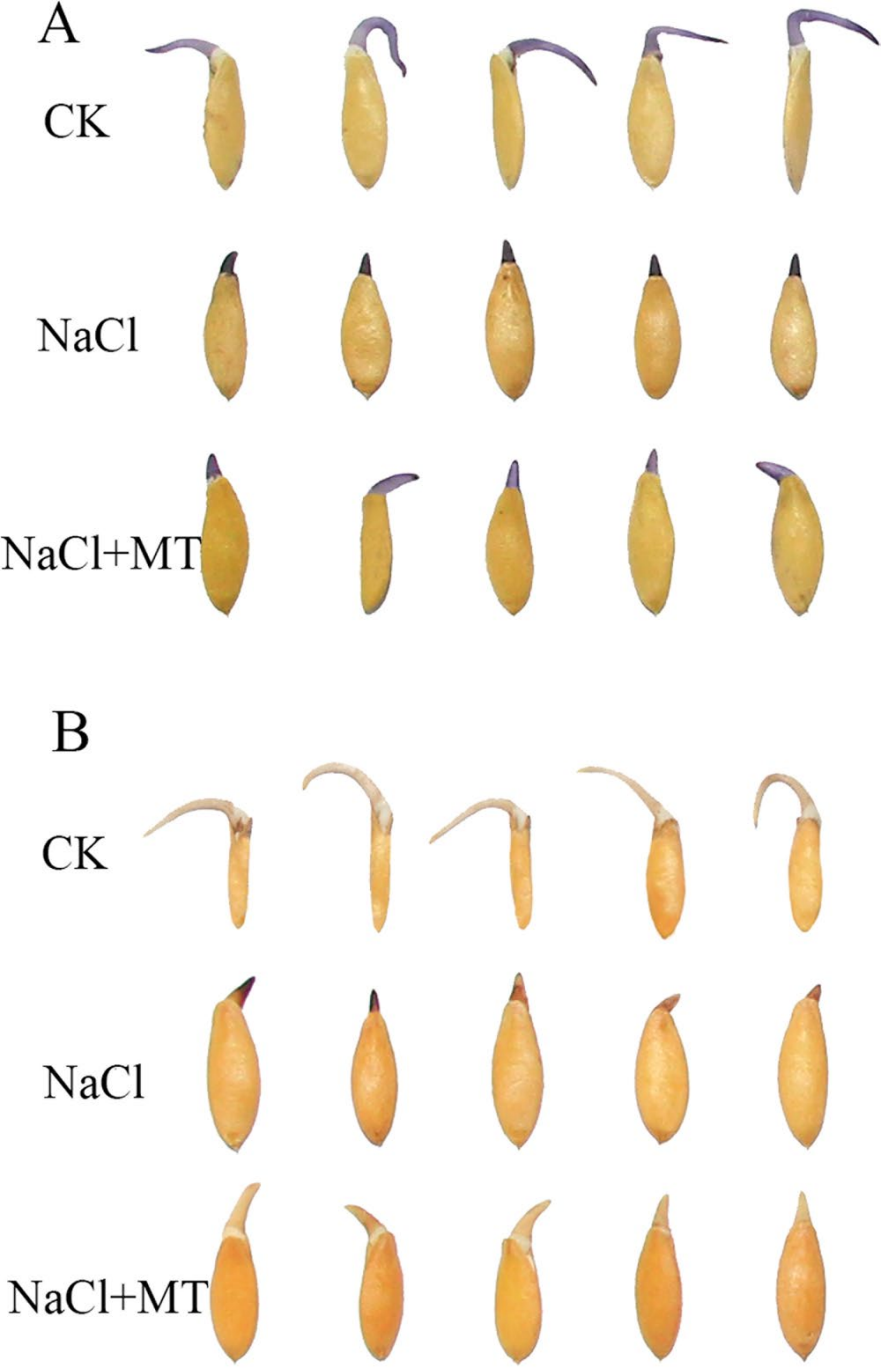
Detection of superoxide anion (**A**) and Hydrogen peroxide (**B**) accumulation in cucumber seeds. NBT reacts with O_2_˙^−^ to form a dark blue insoluble formazan compound. DAB is oxidized by H_2_O_2_ in the presence of peroxidases and produces reddish brown precipitate.

As a major abiotic stress worldwide, high salinity can limit plant growth and inhibit seed germination. Salt stress induces the accumulation of ROS such as O_2_˙^−^, H_2_O_2_, and ·OH^8, 9, 35^. Melatonin has been shown to relieve the inhibitory effect of high salinity on germination of cucumber seeds by scavenging ROS^8^. Exogenous melatonin treatment significantly up-regulated the expression of *CsCu-ZnSOD*, *CsFe-ZnSOD*, *CsPOD*, and *CsCAT* and decreased oxidative damage^8^. During the seed germination, the contents of superoxide anion and H_2_O_2_ were significantly increased under salt stress compared to CK (Fig. 3). Over accumulation of ROS can result in the oxidization of some functional important proteins^36, 37^, and hence impede germination^38^. Melatonin treatment obviously protected the seed germination from the oxidative damages (Fig. 3). Through proteomic analysis, we identified two glutathione peroxidases (GPX, B6DQ61 and B9RCA6), one glutaredoxin (U3RGD2), one superoxide dismutase (SOD, Q6QGY4), and two peroxidase-2-like proteins (POD, Q39650 and Q6UBM4) (Table 1). After exogenous melatonin treatment, the GPXs, SODs, and PODs were up-regulated from 2.1- to 10.2-fold under salt stress. The DAB and NBT staining data and the proteomics results together further complement the existing evidences that melatonin has the ability to regulate the antioxidant enzymes.

14-3-3 proteins are phosphoserine-binding proteins. In plants, they regulate the activities of the plasma membrane H^+^-ATPase and enzymes involved in carbon and nitrogen metabolism. Nevertheless, more and more plant signaling proteins are now being recognized as 14-3-3-interacting proteins^39^. Plant 14-3-3 proteins have roles in regulating plant development and stress responses^39^. For instance, 14-3-3 protein GRF9 is involved in allocating additional carbon from the shoot to the root in response to polyethylene glycol-induced water stress^40^. 14-3-3 proteins have also been identified as important regulators of salt tolerance and as mediators of the Salt Overly Sensitive (SOS) pathway^41^. Here, we found two 14-3-3-like proteins (W9S288 and Q5UFR1) through proteomic analysis. Two of these proteins were up-regulated by melatonin approximately 31.9- and 24.6-fold. This result further confirmed the function of melatonin as a plant growth regulator under salt stress^42^.

### Melatonin regulates the protein synthesis under salt stress

**Figure 4:**
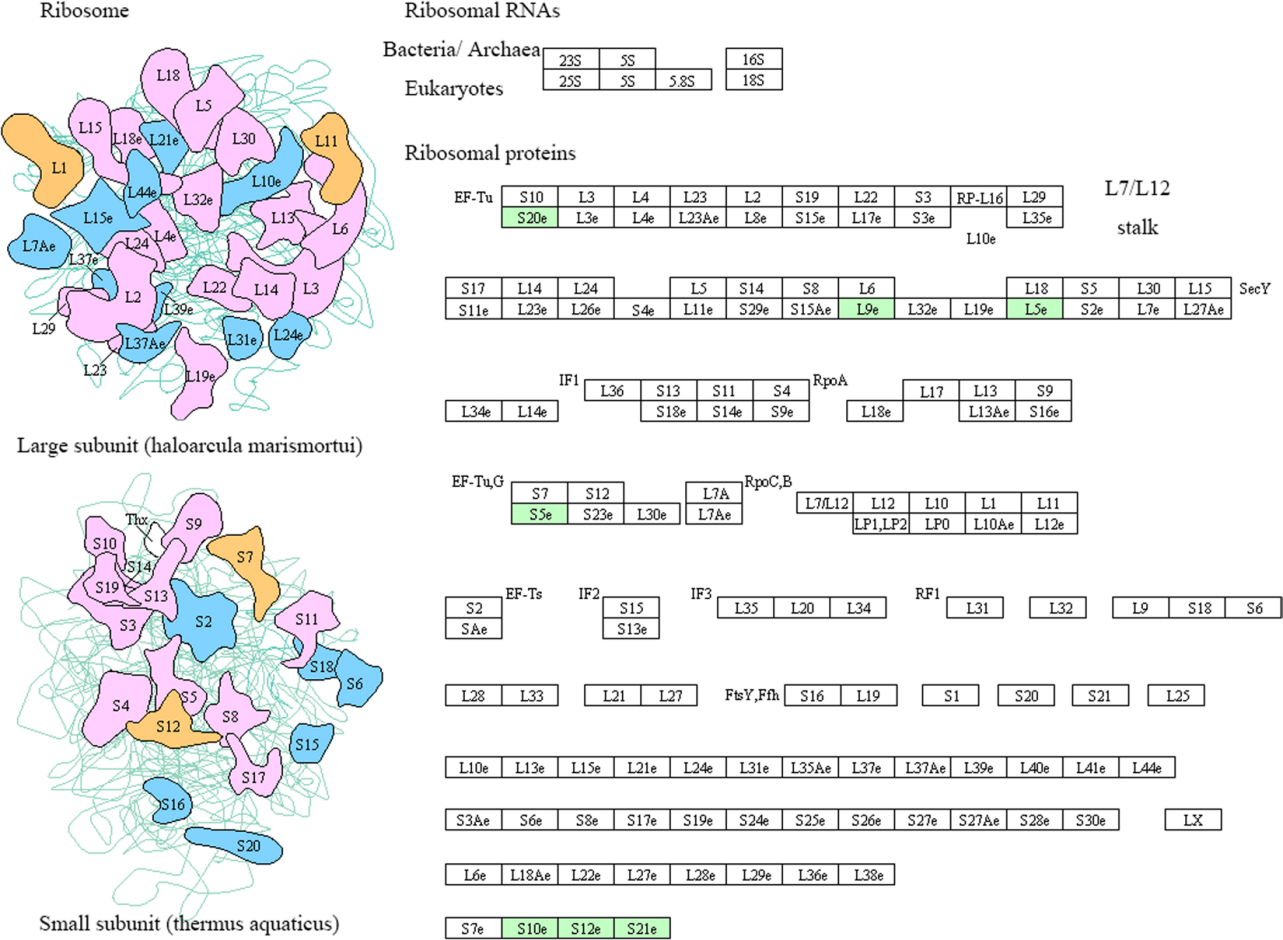
Ribosome pathway obtained from KEGG pathway analysis. The protein names in green color are differentially regulated by melatonin under salt stress during seed germination.

Ribosomes are present in dry seeds, and soon after imbibition, they combine with mRNAs to form polysomes for protein synthesis. Ribosomes consist of a small and a large subunit. Here, the protein levels of one ribosomal large subunit family protein (A0A067KWC5) and five ribosomal small subunit family proteins (W9QS28, A0A067L2F9, A9PAL8, B9RFA5 and O65731) were upregulated by melatonin under salt stress conditions (Table 1 and Fig. 4). These results suggest that melatonin promoted protein synthesis by regulating levels of ribosomal subunits during seed germination under salt stress.

### Melatonin promotes the lipid and starch catabolism under salt stress

**Figure 5:**
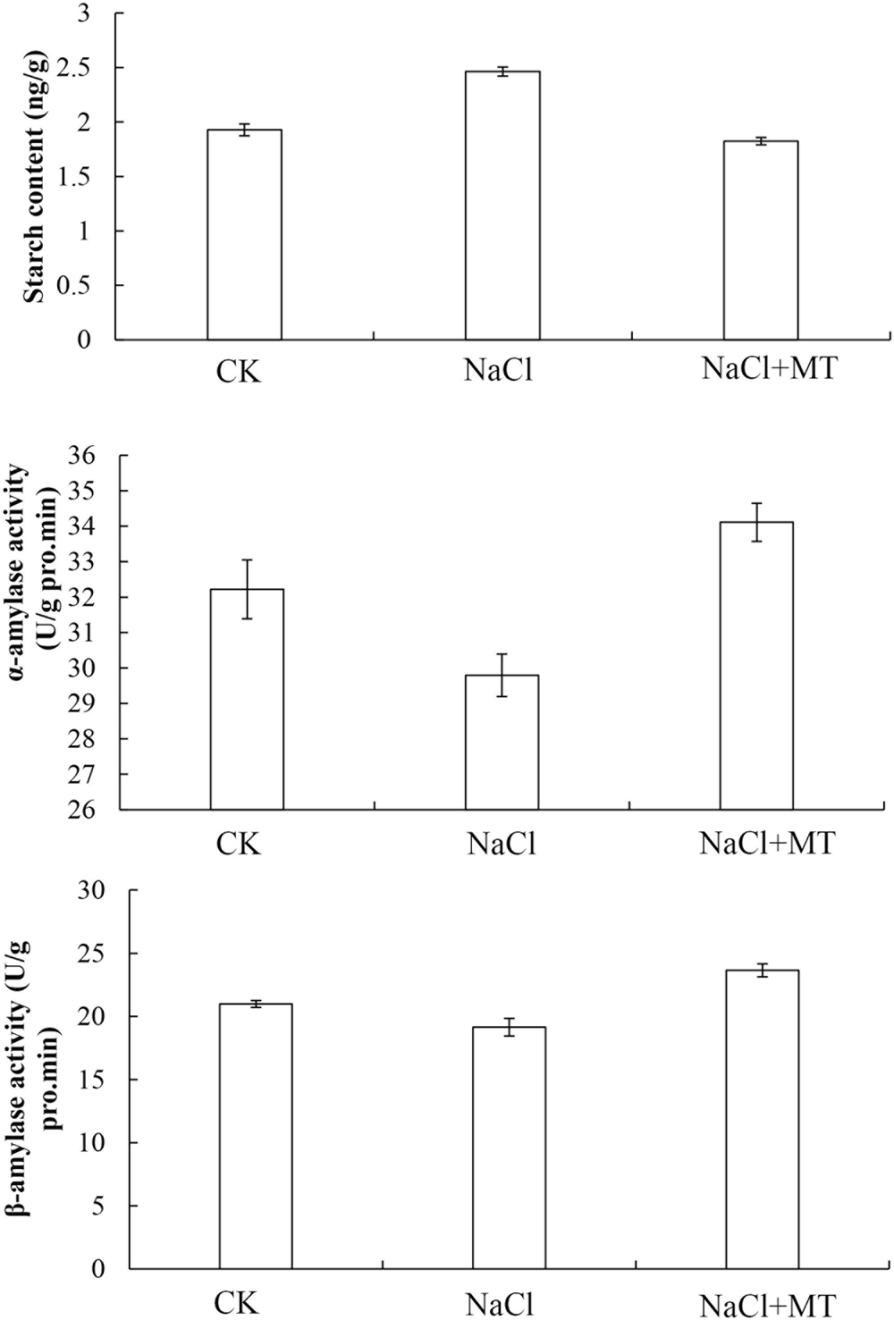
Effects of 1 μM melatonin on starch content (**A**) and α, β-amylase activities (**B**,**C**) during germination of cucumber seeds under salt stress. Vertical bars represent ± S.E (n = 3).

**Figure 6:**
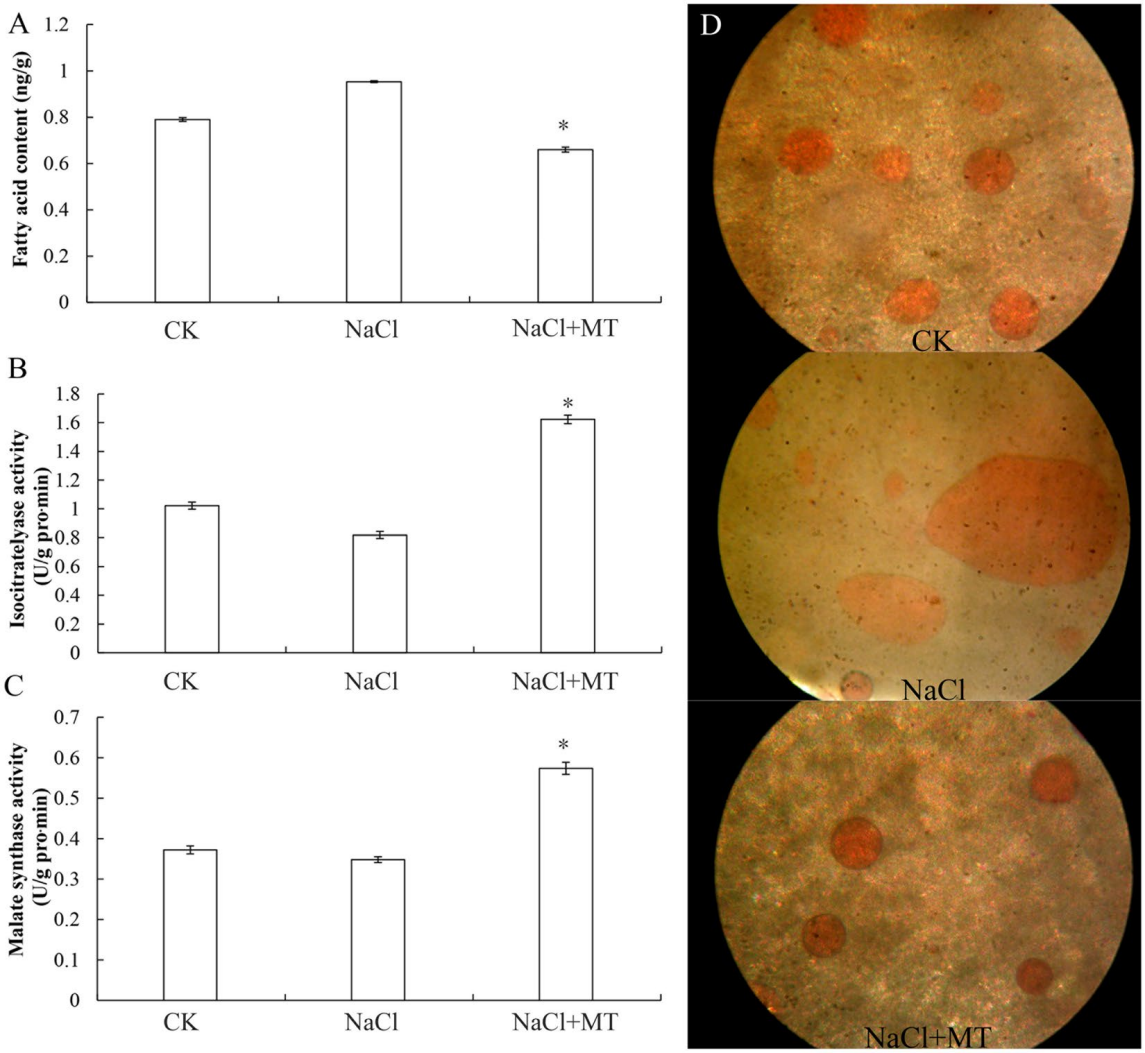
Effects of 1 μM melatonin on lipid content (**A**,**D**) and activities of isocitrarelyase (**B**) and malate synthase (**C**) during germination of cucumber seeds under salt stress. Lipid droplets in (**D**) were studied using 0.5% sudan red staining. Vertical bars represent ± S.E (n = 3).

To examine whether melatonin influenced lipid and starch catabolism, the other two important storage reserves, in cucumber seeds under high salinity, we measured the content of lipid and starch as well as the activities of α- and β-amylase during seed germination (Figs 5 and 6A). Both starch and lipid content in seeds of NaCl treatment were markedly higher than seeds in CK. In the presence of NaCl, the catabolism of starch and lipid during germination was remarkably inhibited and delayed (Figs 5A and 6A). The activities of α- and β-amylase were lower in seeds of NaCl treatment than in CK in the context of salt stress. After exogenous melatonin treatment, the activities of α- and β-amylase were significantly increased 14.5% and 23.5% compared to NaCl treatment, respectively, while the starch content was 34.7% lower than NaCl treatment, indicating a role of melatonin in starch catabolism (Fig. 5B,C).

Lipids are important natural resources because they can be used for energy production. Through β-oxidation and the glyoxylate cycle, lipids are converted to energy for seed germination^43^. We further investigated the activities of two key enzymes, isocitratelyase and malate synthetase, in the glyoxylate cycle (Fig. 6B,C). The data showed that both isocitratelyase and malate synthetase were significantly decreased compared to CK under salt stress by 20.1% and 6.5%, respectively. Melatonin treatment greatly increased the activity of both isocitratelyase and malate synthetase under NaCl stress by 98.5% and 64.9%, respectively. In agreement with this, the lipid content was 30.7% lower upon melatonin treatment compared to NaCl treatment, suggesting a positive effect for melatonin in converting lipids to energy (Fig. 6A). The Sudan red staining visually showed that the degradation of lipid droplets were significantly inhibited by salt stress in cucumber seeds (Fig. 6D). The lipid droplets were large and irregular in shape under salt stress compared to CK. Melatonin treatment greatly increased the degradation of lipid droplets (Fig. 6D). Taken together, these results indicated that melatonin alleviates the inhibition of seed germination caused by NaCl stress by facilitating storage protein, starch, and lipid degradation to supply energy and nutrition for seed germination (Figs 5 and 6).

### Melatonin participates in the regulation of energy production

Glycolysis is an important pathway for energy production in the cytosol of plant cells. A key enzyme involved in this pathway is glyceraldehyde-3-phosphate dehydrogenase (GAPDH), which is indispensable to maintain the balance of cellular ATP levels and carbohydrate metabolism. GAPDH catalyzes the first step of glycolysis by converting D-glyceraldehyde-3-phosphate (G3P) into 3-phospho-D-glyceroyl phosphate or D-glycerate-1, 3-bisphosphate. It has been reported that a putative GAPDH accumulates in germinated rice seeds^44^. Our data showed that 21 proteins involved in carbohydrate metabolism accumulated (Table 1), suggesting that these proteins might be directly associated with cucumber seed germination. Among these proteins, 19 proteins were significantly up-regulated by melatonin under salt stress. Melatonin significantly increased GAPDH (E1B2J6) levels by approximately 5.86-fold under salt stress (Table 1), suggesting that exogenous melatonin regulated energy production during early stages of seed germination. Triose phosphate isomerases (TPI) are involved in several metabolic pathways including glycolysis, gluconeogenesis, and the Calvin cycle^45^. It catalyzes the reversible interconversion of dihydroxyacetone phosphate (DHAP) to glyceraldehyde-3-phosphate (GAP)^46^. An *Arabidopsis* mutant (*pdtpi*) lacking plastid TPI activity showed severely stunted establishment of seedlings and did not reach reproductive maturity^45^. Reduction of plastid TPI activity resulted in the buildup of glycerol, G-3-P, DHAP, and MG, which impaired seedling establishment and chloroplast development^45^. In this article, we found that four hypothetical TPI proteins (A0A072U2W1, B9GJN0, A0A067LKT3, and Q38IW8) were significantly up-regulated by exogenous melatonin under salt stress by an average of 8.13-fold. Some other important proteins involved in glycolysis (enolase 1, A0A067JHW3; fructose-bisphosphate aldolase, A0A067KLE6; phosphoglycerate kinase, A1BQH1, I1MJC7 and B9HY30) were also regulated by melatonin under salt stress. KEGG pathway analysis also supported a role for melatonin in regulating glycolysis (Fig. S2). These results indicated an interaction between glycolysis and the melatonin-mediated salt stress response.

**Figure 7:**
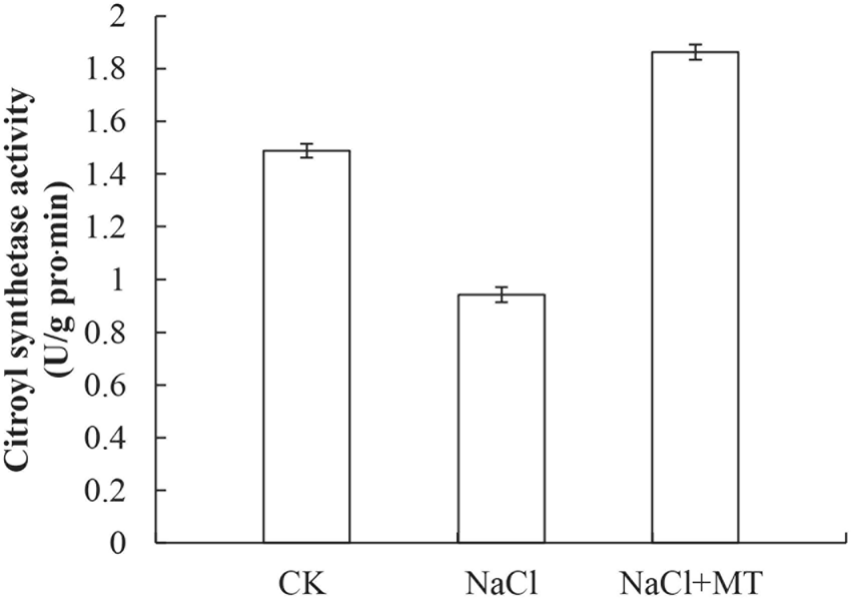
Effects of 1 μM melatonin on citroyl synthetase activity during germination of cucumber seeds under salt stress. Vertical bars represent ± S.E (n = 3).

The citric acid cycle, also known as the tricarboxylic acid (TCA) cycle, is a key metabolic pathway that unifies carbohydrate, fat, and protein metabolism. Through catabolism of carbohydrate, fat, and protein, a two-carbon organic product acetate, in the form of acetyl-CoA, is produced and enters the citric acid cycle to produce energy and reducing power^47^. As shown in Table 1, we identified one isocitrate dehydrogenase (B9GHS2) and one malate dehydrogenase (P17783) through proteomic analysis. After exogenous melatonin treatment, these two TCA cycle enzymes were remarkably increased by 2.03 and 10.62 fold, respectively. This result indicated the involvement of melatonin in regulating catabolism of energy production. To further demonstrate the regulatory effect of melatonin on TCA cycle, we measured the activity of citroyl synthetase, another key enzyme in the TCA cycle (Fig. 7). Our data showed that NaCl stress significantly inhibited citroyl synthetase activity by approximately 12.3% compared to CK. In contrast, melatonin treatment showed a significant reversal of NaCl-induced inhibition of citroyl synthetase activity (increased by 60.1% compared to NaCl treatment) (Fig. 7). KEGG pathway analysis also supported a role for melatonin in regulating the TCA cycle and energy production (Figs S3 and 4). These results demonstrated that melatonin was involved in regulating the TCA cycle and promoted energy catabolism during seed germination under salt stress.

## Discussion

Seed germination is a complex and crucial process that determines seedling establishment. Germination includes a series of physical and metabolic events^48^. As a major abiotic stress worldwide, high salinity can limit plant growth and inhibit seed germination. In this study, NaCl stress significantly decreased the germination rates of cucumber seeds (Fig. 1). The inhibitory effect of high salinity on cucumber seed germination was alleviated by the application of 1 μM melatonin (Fig. 1). A protective role of melatonin in cucumber seed germination response to NaCl stress was observed.

Storage proteins are synthesized during the late stage of seed development and deposited in protein storage vacuoles in mature seeds^49^. During seed germination, these proteins are mobilized to provide nutrients for seed germination and subsequent seedling growth^50^. In our study, during germination of cucumber seeds under salt stress, storage protein showed an increasing abundance after melatonin treatment, including two globulin-like proteins (P13744 and Q8W1C2) and two vicilin-like proteins (Q39651 and Q9ZWI3) (Table 1). Our data showed that melatonin might have the ability to regulate the storage proteins catabolism under salt stress.

Cortical microtubules (such as tubulin and actin) are associated with cell division. It showed high accumulation in the radicle of tomato and cucumber during seed germination^30^. Han *et al*. observed the accumulation of tubulin after 24 h imbibition in rice embryo^51^. Here, we found that exogenous melatonin significantly increased the abundance of hypothetical tubulin *β* chain (B9S382) and actin 7 (A0A067JQD9) (Table 1) during seed germination, showing that melatonin plays an important role in cell division and elongation during seed germination.

Over accumulation of ROS can result in oxidative stress. Reducing oxidized proteins is another critical way for plants to cope with ROS. Upon imbibition, ROS contents increased gradually. ROS can be scavenged efficiently by the antioxidant enzymes such as superoxide dismutases, glutathione S-transferase, and peroxidases^52^. Many of these redox regulation proteins were identified during germination^25^. Proteomic analysis showed that exogenous melatonin treatment significantly up-regulated the level of antioxidant enzymes, including two glutathione peroxidases (B9RCA6 and B6DQ61), one superoxide dismutase (Q6QGY4), two peroxidase-2-like proteins (Q39650 and Q6UBM4) and two peroxiredoxins (A9P8D8 and B9HII6) (Table 1). In response to NaCl stress-induced ROS, melatonin stimulates the activity of major antioxidant enzymes under adverse conditions. Melatonin treatment obviously protected the seed germination from the oxidative damages (Fig. 3). The similar protective role of melatonin in responded to leaf senescence in *Malus hupehensis* were observed^17^. Three glutathione peroxidases (GPX), one glutathione reductase (GR), two ascorbate peroxidases (APX), and one L-galactose dehydrogenase were regulated by melatonin during leaf senescence. Under H_2_O_2_ stress, four peroxidase superfamily proteins and an ascorbate peroxidase (APX) were significantly increased by melatonin^18^. This result is consistent with previous reports at the transcriptome level that melatonin increases the expression level of corresponding genes to protect cells^53^.

Melatonin also regulated the abundance of proteins involved in cellular response to stress. Stress responsive proteins, including HSPs and late embryogenesis abundant (LEA) proteins, accumulated during seed maturation^24, 54^. As molecular chaperones, HSPs can repair stress-damaged proteins and help protect cells against stress^32, 33^. Interestingly, we found that 13 HSPs (G7L007, M5W6U5, B9HDE5, W9RXY8, Q9M4E7, A0A067JRU1, A0A067KUJ3, B9RMP5, H6TB40, A0A072UYS2, P19243, H6TB44, and H6TB46) (Table 1) were significantly up-regulated by melatonin under NaCl stress. This indicates the involvement of HSPs in the melatonin-mediated NaCl stress response. As a plant growth regulator, melatonin regulated two 14-3-3 family proteins (W9S288 and Q5UFR1) (Table 1) to alleviate the inhibitory effects of NaCl stress. It has been reported that nearly all 14-3-3 protein–protein interactions could be regarded as being involved in signaling at some level. 14-3-3 proteins affect the activity of various enzymes and ion channels of central importance in plant biochemistry^55–57^. Therefore, the relationship between melatonin and 14-3-3 proteins still requires further investigation.

Once the dry seeds uptake water, mRNAs and ribosomes assemble rapidly into polysomes, then immediately begin to synthesize proteins with other components^58^. In Bermuda grass, the protein levels of 12 ribosomal large subunit family proteins and 8 ribosomal small subunit family proteins were significantly regulated by melatonin under oxidative stress conditions^18^. In this study, melatonin up-regulated one ribosomal large subunit family protein (A0A067KWC5) and five ribosomal small subunit family proteins (W9QS28, A0A067L2F9, A9PAL8, B9RFA5 and O65731) under salt stress, indicating a role of melatonin in protein synthesis (Table 1 and Fig. 4).

Seed germination and subsequent seedling growth require a large amount of energy and carbon skeletons^16, 22^. Energy production plays key role in whole seed germination. After absorbing plenty of water, the amino acid metabolism, glycolysis, and the TCA cycle are activated in seeds. We identified several proteins that were involved in energy production. Proteomic data showed that melatonin regulated several metabolic pathways, including glycolysis, the citric acid cycle, and the glyoxylate cycle. From the catabolism of starch and lipid to energy production, many enzymes involved in these pathways were observed changing in abundance (Figs 5 and 6). Starch is considered to be the main source of ATP^24, 59^. Melatonin up-regulated the activities of relevant enzymes (α, β- amylase) and promoted starch catabolism for ATP production (Fig. 5). Melatonin also promoted lipid catabolism and up-regulated activities of two key enzymes (isocitratelyase and malate synthetase) of glyoxylate cycle (Fig. 6). Glycolysis is one pathway for formation of ATP during germination. TCA cycle-related proteins such as aconitate hydratase, dihy drolipoyl dehydrogenase 1, and alpha-glucan phosphorylase were up-regulated during seed germination^14^. The important function of glycolysis and TCA cycle is to provide energy for cell activity and to supply carbon skeleton for macromolecule biosynthesis during seed germination^60^. It has been proposed that energy production is an early key event for seed germination^16, 22, 49^. In this study, enzymes involved in glycolysis (E1B2J6, A0A072U2W1, B9GJN0, A0A067LKT3, Q38IW8, A0A067KLE6, I1MJC7, A1BQH1, B9HY30, and A0A067JHW3), the citric acid cycle (B9GHS2, P17783 and citroyl synthetase) (Fig. 7), and the glyoxylate cycle (isocitratelyase and malate synthetase) were significantly regulated by melatonin (Table 1). These results fully demonstrated that melatonin could be considered as a positive regulatory factor in energy metabolism, which is consistent with previous reports^42^.

## Conclusion

**Figure 8:**
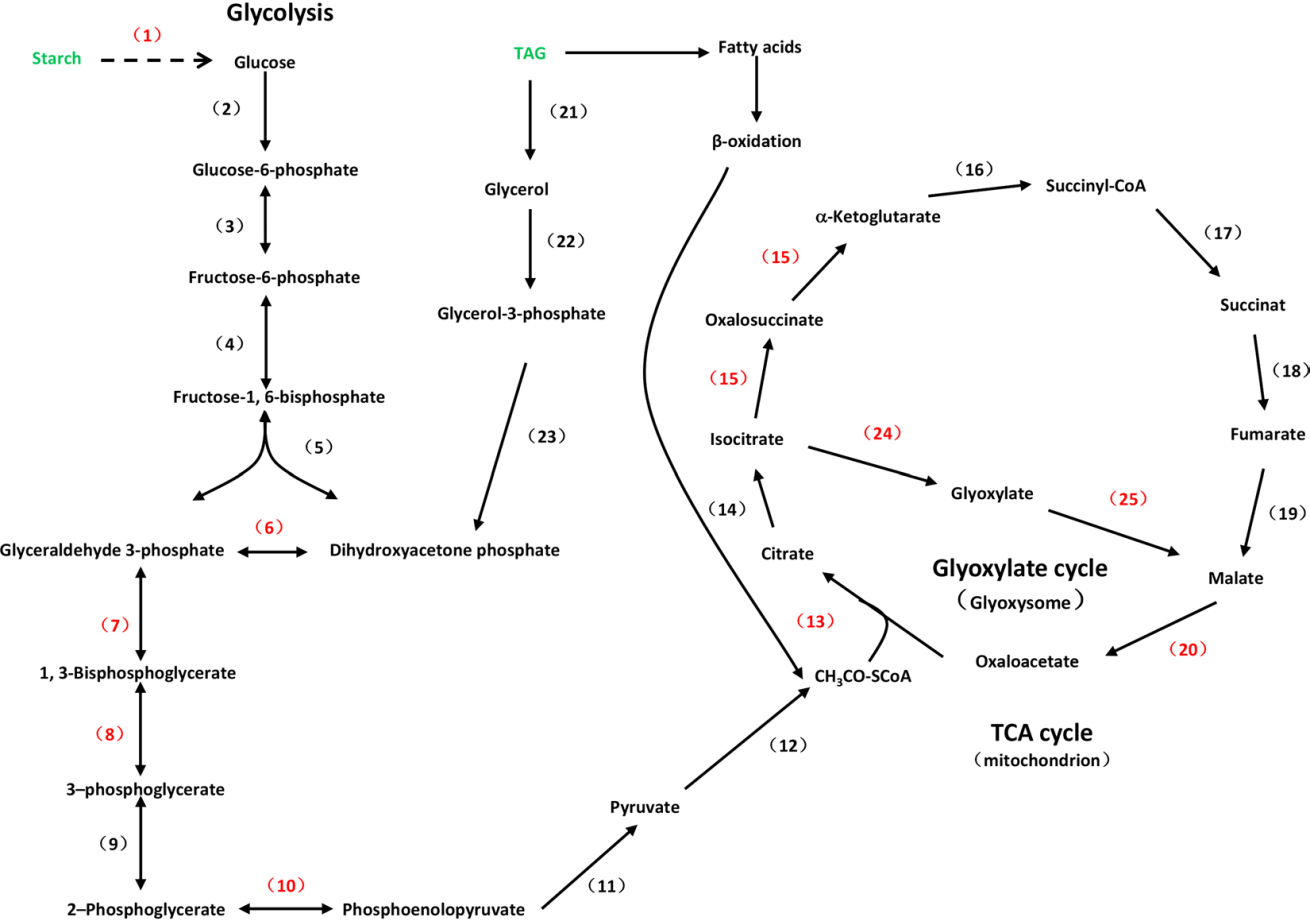
Schematic representation of energy metabolism that melatonin alleviated the inhibitory effects of NaCl stress on seed germination. Glycolysis, TCA cycle and glyoxylate cycle were regulated by melatonin. The arrows represent the direction of the chemical reaction. The Arabic numerals or molecules in red and green are up and down regulated by melatonin, respectively. (1) α, β-amylase (2) hexokinase (3) phosphoglucose isomerase (4) phosphofructokinase (5) aldolase (6) triosephosphate isomerase (7) glyceraldehyde 3-phosphate dehydrogenase (8) phosphoglycerate kinase (9) phosphoglycerate mutase (10) enolase (11) pyruvate kinase (12) pyruvate dehydrogenase (13) citrate synthase (14) aconitase (15) isocitrate dehydrogenase (16) ketoglutarate dehydrogenase (17) succinyl-coa synthetase (18) succinate dehydrogenase (19) fumarase (20) malate dehydrogenase (21) tag lipases (22) glycerol kinase (23) glycerol phosphate dehydrogenase (24) isocitrate lyase (25) malate synthase.

Our previous publications show that melatonin alleviates abiotic stress and promotes seed germination^8, 9^, but the regulatory roles of melatonin during seed germination remained unclear at the proteomic level. In this study, exogenous melatonin acted as a regulatory factor for proteins involved in the stress response, cell elongation, glycolysis, the citric acid cycle, and the glyoxylate cycle (Fig. 8). Melatonin relieves the inhibitory effect of high salinity on the germination of cucumber seeds mainly by regulating energy production. Indeed, this study is another step toward establishing an unequivocal role for melatonin in promoting seed germination under NaCl stress at the proteomic level.

## Methods

### Germination conditions

Cucumber seeds were soaked in distilled water, and 1 μM melatonin solution for 24 hr, sterilized in 5% sodium hypochlorite solution for 10 min, and rinsed in distilled water five times. To examine the effects of salt stress on germination, water-pretreated cucumber seeds were placed in Petri dishes (13 × 13 cm) containing filter paper (Whatman International Ltd, Maidstone, UK) at 28 °C for 24 hr in a growth chamber in the dark. The filter paper was soaked with 15 ml of distilled water (control) or 150 mM NaCl solution. Seeds were considered to be germinated when the seed coat was broken and a radicle was visible. In view of the initial and rapid germination under NaCl stress at 12 and 14 hr, respectively, the seeds were sampled at 0, 12, 14, 16, 18, and 24 hr after germination for each treatment. All samples were rapidly frozen in liquid nitrogen and stored at −80 °C for analysis. CK: water-pretreated seeds germinated under water, NaCl: water-pretreated seeds germinated under 150 mM NaCl solution, NaCl + MT: 1 μM melatonin-pretreated seeds germinated under 150 mM NaCl solution. Each treatment contained 500 seeds. All experiments were conducted in triplicate.

### Determining the content of starch, lipid, and relevant enzymes involved in glycolysis, the citric acid cycle, and the glyoxylate cycle by enzyme-linked immunosorbent assay (ELISA)

Approximately 0.5 g of fresh seeds were weighed and homogenized in 2 ml of precooled PBS. Extracts were centrifuged at 4000 rpm for 20 min. The liquid supernatant was collected and stored at −20 °C for analysis by enzyme-linked immunosorbent assay (ELISA). The ELISA procedures were conducted according to the instructions provided by the manufacturer (China Agricultural University, Beijing, China). Lipid droplets in cucumber seeds were studied using 0.5% Sudan red staining.

### Histochemical detection of superoxide and H_2_O_2_

Histochemical detection of H_2_O_2_ and superoxide anion in cucumber seeds was detected as described previously^61^. Briefly, cucumber seeds were collected at 14 h after imbibition and soaked in 3, 3ʹ-Diaminobenzidine (DAB, 1 mg/ml, pH 7.5) solution at room temperature for 1 h. The appearance of reddish brown precipitate was monitored to indicate the accumulation of H_2_O_2_.

Superoxide anion was detected by using Nitrotetrazolium blue chloride (NBT) as the chromogenic substrate. Seeds were incubated in 0.2% NBT in 50 mM sodium phosphate buffer (pH 7.5) at room temperature for 1 h. The accumulation of superoxide anion was determined by the visualization of dark-blue color.

### Total protein extraction

Total protein was extracted from cucumber seeds as described previously^14, 61, 62^, with some modifications. Samples (~3 g) were ground in liquid nitrogen, and the resulting powders were precipitated in trichloroacetic acid (TCA)/acetone (1:9 w/v) solution for 15–18 hr at −20 °C. The samples were then centrifuged at 7000 × g for 30 min at 4 °C, the supernatant was discarded, and the precipitate was rinsed three times with 1 ml chilled (−20 °C) acetone. The pellet was dried at room temperature and dissolved in an appropriate volume of extraction buffer (4% SDS, 1 mM DTT, 150 mM Tris-HCl, pH 8). After a 3-min incubation in boiling water, the homogenate was sonicated on ice. The crude extract was then incubated in boiling water again and clarified by centrifugation at 16,000 × g at 25 °C for 30 min. Total protein content was determined using a BCA Protein Assay Kit (Bio-Rad, USA).

### Protein Digestion

Digestion of protein (250 μg for each sample) was performed according to the FASP procedure described by Wisniewski, Zougman *et al*.^63^. Briefly, the detergent, DTT and other low-molecular-weight components were removed using 200 μl of UA buffer (8 M urea, 150 mM Tris-HCl, pH 8.0) by repeated ultrafiltration (Microcon units, 30 kD) facilitated by centrifugation. Then, 100 μL of 0.05 M iodoacetamide in UA buffer was added to block reduced cysteine residues, and the samples were incubated for 20 min in the dark. The filtrate was washed with 100 μl of UA buffer three times and then 100 μl of 25 mM NH_4_HCO_3_ twice. Finally, the protein suspension was digested with 3 μg of trypsin (Promega) in 40 μl of 25 mM NH_4_HCO_3_ overnight at 37 °C, and the resulting peptides were collected as a filtrate. The peptide content was estimated by UV light spectral density at 280 nm using an extinction coefficient of 1.1 of a 0.1% (w/v) solution, which was calculated on the basis of the frequency of tryptophan and tyrosine in vertebrate proteins.

### Liquid Chromatography (LC) - Electrospray Ionization (ESI) Tandem MS (MS/MS) Analysis by Q Exactive

The peptides from each sample were desalted on C18 Cartridges (Empore™ SPE Cartridges C18, bed I.D. 7 mm, volume 3 ml, Sigma), then concentrated by vacuum centrifugation, and reconstituted in 40 µl of 0.1% (v/v) trifluoroacetic acid. MS experiments were performed on a Q Exactive mass spectrometer that was coupled to Easy nLC (Proxeon Biosystems, now Thermo Fisher Scientific). Each sample (5 μg of peptide) was loaded onto a C18-reversed phase column (Thermo Scientific Easy Column, 10 cm long, 75 μm inner diameter, 3 μm resin) in buffer A (2% acetonitrile and 0.1% formic acid) and was separated with a linear gradient of buffer B (80% acetonitrile and 0.1% formic acid) at a flow rate of 250 nL/min controlled by IntelliFlow technology over 120 min. MS data were acquired using a data-dependent top10 method dynamically choosing the most abundant precursor ions from the survey scan (300–1800 m/z) for HCD fragmentation. Determination of the target value was based on predictive Automatic Gain Control (pAGC). Dynamic exclusion duration was 25 s. Survey scans were acquired at a resolution of 70,000 at m/z 200 and resolution for HCD spectra was set to 17,500 at m/z 200. The normalized collision energy was 30 eV, and the underfill ratio, which specifies the minimum percentage of the target value likely to be reached at maximum fill time, was defined as 0.1%. The instrument was run with peptide recognition mode enabled. MS experiments were performed in triplicate for each sample.

### Sequence Database Searching and Data Analysis

The MS data were analyzed using MaxQuant software version 1.3.0.5. MS data were searched against the UniProtKB Escherichia coli database (2585998 total entries, downloaded 06/07/12). An initial search was set at a precursor mass window of 6 ppm. The search followed an enzymatic cleavage rule for Trypsin/P and allowed a maximum of two missed cleavage sites and a mass tolerance of 20 ppm for fragment ions. Carbamidomethylation of cysteines was defined as a fixed modification, while protein N-terminal acetylation and methionine oxidation were defined as variable modifications for database searching. Label-free quantification was carried out in MaxQuant as previously described^2^. To control the number of false positive identifications among all identifications found by a peptide identification search, the false discovery rate was set (FDR ≤ 0.01). Intensity-based absolute quantification (iBAQ) in MaxQuant was performed on the identified peptides to quantify protein abundance.

### Statistical analysis

All data were analyzed by one-way ANOVA using Duncan's multiple range tests (P < 0.05). The experiment had a completely randomized design. All values reported in this study are the means of three replicates.

## Electronic supplementary material

Table s1, Figs1-s4

Supplementary File 1
