# Supplementary material for: Proteomic analysis reveals a role of melatonin in promoting cucumber seed germination under high salinity by regulating energy production
Source: Sci Rep. 2017 Mar 29;7:503. doi: 10.1038/s41598-017-00566-1 (PMC5428666; doi:10.1038/s41598-017-00566-1)
Supplement: Supplementary file 1 — Table s1, Figs1-s4 [file 41598_2017_566_MOESM1_ESM.pdf]

Proteomic analysis reveals a role of melatonin in promoting cucumber seed germination under high salinity by regulating energy production

Na Zhang[1*], Hai-Jun Zhang[1, 2*], Qian-Qian Sun[1*], Yun-Yun Cao[1], Xing-Sheng Li [3], Bing Zhao[1], Ping Wu[2] & Yang-Dong Guo[1]

*These authors contributed equally to this work
[1]College of Horticulture, China Agricultural University, Beijing, China.
[2] Beijing Vegetable Research Center, Beijing Academy of Agriculture and Forestry Sciences, National Engineering Research Center for Vegetables, Beijing 100097, China.
[3] Shandong Provincial Key Laboratory of Cucurbitaceae Vegetable Biological Breeding, Shandong Huasheng Agriculture Co. Ltd, Shandong, China
Corresponding author: Y.-D. Guo, College of Horticulture, China Agricultural University, No. 2, Yuanmingyuan West Rd, Beijing 100193, P. R. China.
Tel: (86-10)-62734845; Fax: (86-10)-62733404; E-mail: yaguo@cau.edu.cn.
Co-Corresponding author: P. Wu, Beijing Vegetable Research Center, Beijing Academy of Agriculture and Forestry Sciences, No. 50, Zhanghua Rd, Beijing 100097, P. R. China.
Tel: (86-10)-51503077; E-mail: wuping@nercv.org
B. Zhao, College of Horticulture, China Agricultural University, No. 2, Yuanmingyuan West Rd, Beijing 100193, P. R. China.
Tel: (86-10)-62734095; Email: zhaobing@cau.edu.cn

Table. S1 Differentially expressed proteins by melatonin and NaCl stress effects in cucumber seeds.

		(NaCl+MT)/NaCl		NaCl /CK	
Protein ID	Description	Value	*P*	Value	*P*
Q5UFR1	14-3-3 protein	24.64	0.00000	-	-
W9S288	14-3-3-like protein gf14 iota	31.92	0.00004	-	-
Q39558	14-3-3-like protein gf14 omega	0.66	0.22418	-	-
B9HII6	1-cys peroxiredoxin	2.17	0.00651	5.19	0.00138
M5W204	26s proteasome non-atpase regulatory subunit 4 homolog	2.10	0.00011	8.42	0.00427
I1K657	26s proteasome regulatory subunit rpn13-like isoform x1	3.72	0.00531	-	-
G7IFI0	28 kda heat- and acid-stable phosphoprotein	0.19	0.00201	0.59	0.13080
W9QS28	40s ribosomal protein s10-like	10.59	0.00008	-	-
A0A067L2F9	40s ribosomal protein s12-like isoform x2	6.60	0.00005	-	-
A9PAL8	40s ribosomal protein s20-2	4.85	0.00001	4.40	0.00023
B9RFA5	40s ribosomal protein s21-2	9.81	0.00139	-	-
O65731	40s ribosomal protein s5	43.20	0.00061	-	-
M5WFB9	5-methyltetrahydropteroyltriglutamate--homocysteine methyltransferase	12.42	0.00693	-	-
Q6UNT2	60s ribosomal protein l5	0.32	0.00645	0.56	0.29084
A0A067KWC5	60s ribosomal protein l9	6.20	0.00036	-	-
A0A067JQD9	Actin-7	19.51	0.00010	2.40	0.00833
Q0GYN5	Acyl- -binding protein	0.14	0.00005	-	-
W9SCA0	Acyl- -binding protein	0.26	0.00462	-	-
C6TKB1	Adenylate kinase 4	26.42	0.00001	-	-
W9SB70	Alpha-xylosidase 1-like	6.36	0.00003	7.26	0.00008
O04057	Aspartic proteinase	4.09	0.00003	15.40	0.00009
Q5QCG3	Atp synthase f1 subunit 1	-	-	2.50	0.01669
B9T1V8	Atp synthase subunit mitochondrial-like	11.33	0.00001	3.76	0.00007
A9PCU6	Calcium-binding ef hand family protein	0.12	0.00002	0.16	0.00000
G7L3N5	Calmodulin	4.20	0.00001	-	-
B9N6T6	Calmodulin-7	0.20	0.00348	0.28	0.00656
M5WAL0	Calmodulin-7	0.18	0.00006	0.32	0.00492
V7BGM6	Calmodulin-like protein 11	-	-	2.65	0.00050
I1NF03	Calreticulin	0.54	0.00368	0.49	0.03150
M5XFT0	Cap-gly domain-containing linker protein 1	0.43	0.00210	0.61	0.13236
W9RHE5	Cap-gly domain-containing linker protein 1	0.29	0.00471	0.59	0.10874
B9SFS0	Carboxymethylenebutenolidase homolog	5.07	0.00028	4.41	0.00323
B9T0Z2	Carrier protein mitochondrial	37.22	0.00003	-	-
M5VVX1	Cd2 antigen cytoplasmic tail-binding protein 2 isoform x1	0.49	0.01656	-	-
G7K984	Corticosteroid 11-beta-dehydrogenase	6.95	0.00029	-	-
Q96403	Cucumber peeling cupredoxin-like	0.45	0.00022	-	-
B9GHC4	Cysteine proteinase rd21a-like	-	-	1.51	0.21080
E5GBR6	Cysteine synthase	7.60	0.00009	-	-
G7KAG6	Cytochrome c oxidase subunit 6b-1-like	0.53	0.00599	-	-
B9HY30	Cytosolic phosphoglycerate kinase family protein	10.79	0.02090	19.00	0.05771

G7JB35	Dead-box atp-dependent rna helicase 56	-	-	2.54	0.02692
K7MB89	Desiccation-related protein pcc13-62	3.32	0.00759	-	-
I1J4C8	Duf3456 domain protein	0.37	0.00851	-	-
P13744	Edestin 2	19.40	0.00001	21.82	0.00001
V5IV18	Elongation factor 1-alpha	9.25	0.00049	4.08	0.00098
U5FMF5	Embryonic protein dc-8	0.32	0.00211	-	-
I1MC31	Endoplasmin homolog isoform x1	0.49	0.00683	-	-
A0A067JHW3	Enolase	6.84	0.00003	9.15	0.00003
M5WBU5	Eukaryotic translation initiation factor 3 subunit j-like	0.17	0.00059	0.47	0.03185
V7AU18	Far upstream element-binding protein 1-like	0.20	0.00018	0.25	0.00470
M5XJW9	Far upstream element-binding protein 2	0.15	0.00068	0.16	0.02009
W9QFK5	Fasciclin-like arabinogalactan protein 1	0.44	0.00098	0.46	0.00014
U5G711	Fasciclin-like arabinogalactan protein 17	11.16	0.00000	1.58	0.00689
M5WXE6	Fasciclin-like arabinogalactan protein 7	2.36	0.00964	-	-
A0A067KAC0	Fasciclin-like arabinogalactan protein 8	0.40	0.00079	-	-
A0A067KLE6	Fructose-1 family protein	2.33	0.00046	3.20	0.00034
I1LZG1	Fructose-bisphosphate cytoplasmic isozyme-like	2.38	0.01167	6.15	0.00003
W9RG25	Glucan endo- -beta-glucosidase 4	0.19	0.00031	0.29	0.04885
C6TG21	Glucan endo- -beta-glucosidase-like protein	0.47	0.00819	0.61	0.00022
A0A067JL19	Glucose and ribitol dehydrogenase homolog 1	13.81	0.00002	13.81	0.00002
U3RGD2	Glutaredoxin-like protein	0.48	0.00718	-	-
I3SE57	Glutathione s-transferase dhar2	-	-	2.17	0.07598
E1B2J6	Glyceraldehyde-3-phosphate dehydrogenase	8.37	0.00003	-	-
B9H3B0	Glycosyl hydrolase family 17 family protein	2.19	0.01513	0.30	0.02392
B7SHL2	Gly-rich rna-binding protein	-	-	2.31	0.01158
I3T6C4	Grf1-interacting factor 3-like	0.20	0.00133	0.32	0.07447
A0A067L6A9	Grpe protein mitochondrial	0.27	0.02822	-	-
B9HJ80	H+-transporting two-sector atpase family protein	-	-	4.51	0.00427
A0A067KUJ3	Heat shock 70 kda mitochondrial	3.06	0.00016	-	-
G7L007	Heat shock 70 kda protein	10.95	0.00003	-	-
M5W6U5	Heat shock 70 kda protein	2.22	0.00015	3.24	0.00004
B9HDE5	Heat shock 70 kda protein 17-like	2.72	0.00006	-	-
W9RXY8	Heat shock cognate protein 80	3.86	0.00007	-	-
Q9M4E7	Heat shock protein 70	11.16	0.00619	10.99	0.03789
B9GVM4	Heat shock protein 70 cognate	-	-	2.15	0.00333
A0A067JRU1	Heat shock protein 83	5.01	0.00009	1.63	0.11292
A0A067JMD9	Histone acetyltransferase	8.36	0.00008	-	-
K7KM74	Histone chaperone domain chz protein	0.56	0.00609	0.46	0.01164
A0A067KRB3	Histone deacetylase hdt1-like	2.46	0.00074	5.15	0.00316
B9ILZ3	Histone h4	26.72	0.00001	2.07	0.00521
Q4ZH67	Hmg1 2-like protein	8.41	0.00010	9.16	0.00006
A0A067KV00	Hsp70 nucleotide exchange factor fes1-like	0.16	0.00053	0.31	0.01468
I1J9X8	Hsp70-hsp90 organizing protein 3	0.25	0.04886	0.59	0.29238
I1K0K7	Hsp70-hsp90 organizing protein 3-like	0.18	0.00009	0.50	0.00470

B9RCY9	Hyaluronan mrna binding family isoform 1	0.17	0.00020	0.57	0.01678
I1KW34	Ice-like protease p20 domain protein	7.84	0.00001	-	-
B9GHS2	Isocitrate dehydrogenase	2.03	0.01631	2.03	0.01631
I1JJD4	Isoform x1	0.43	0.00202	0.61	0.00039
A0A072UYS2	Kda class i heat shock protein	5.34	0.00010	15.67	0.00119
P19243	Kda class i heat shock protein	16.89	0.00000	26.26	0.00005
H6TB44	Kda class ii heat shock	19.34	0.00772	-	-
H6TB46	Kda class iv heat shock protein	9.24	0.02695	-	-
Q7XBA7	Late embryogenesis abundant protein	-	-	4.92	0.00300
E5GC45	Late embryogenesis abundant protein d-29	-	-	2.98	0.12796
B9HV45	Late embryogenesis abundant protein d-34-like	0.01	0.00078	0.03	0.01265
W9R0U6	Late embryogenesis abundant protein d-34-like	0.17	0.00117	0.27	0.00126
A0A067JS88	Late seed maturation protein	0.61	0.11891	2.27	0.14429
Q8W1C2	Legumin a	19.88	0.00000	13.65	0.00002
A0A072UBU4	Leucine-rich repeat extensin-like protein 4	0.13	0.00601	0.22	0.03904
Q9M4E8	Luminal-binding protein 5	0.17	0.00002	0.34	0.00235
A0A067KKK4	Lysm domain-containing gpi-anchored protein 1	0.26	0.00046	0.41	0.00302
I1JZ72	Lysm domain-containing gpi-anchored protein 1-like	0.27	0.00077	0.35	0.00152
A0A075CA98	Lysosomal alpha-mannosidase isoform x1	4.21	0.00017	4.33	0.00378
P17783	Malate dehydrogenase mitochondrial	10.62	0.00000	7.74	0.00000
B9R9D3	Membrane steroid-binding protein 2-like	0.11	0.00001	0.14	0.00962
C6SXJ2	Multiprotein-bridging factor 1a	0.37	0.00123	0.51	0.09015
I3SYP3	Nad -binding rossmann-fold protein	3.68	0.00062	6.08	0.00035
A9PA21	Nascent polypeptide-associated complex subunit alpha-like protein 1	2.05	0.03171	4.12	0.03137
W9RZ99	Nicotinamide mononucleotide adenylyltransferase	5.30	0.00002	3.68	0.00001
A0A067JF59	Nucleoside diphosphate kinase 1	4.30	0.00672	-	-
L7S218	Nucleoside diphosphate kinase b	3.68	0.00266	6.96	0.00096
E5GBW3	Nucleoside diphosphate kinase chloroplastic	7.03	0.00019	-	-
A0A067KFU5	Nucleosome assembly protein 1 3	0.38	0.00030	-	-
B9GI54	Oleosin kda-like	2.32	0.00001	-	-
Q84T21	Oleosin kda-like	2.46	0.00008	2.72	0.01578
K4KD17	Atpb	25.43	0.00028	3.78	0.00271
Q52UN0	Peptidyl-prolyl cis-trans isomerase	5.67	0.00000	9.47	0.00000
Q84TK9	Peptidyl-prolyl cis-trans isomerase family protein	9.96	0.00097	-	-
Q39650	Peroxidase 2-like	3.08	0.01516	-	-
Q6UBM4	Peroxidase 2-like	2.94	0.00001	0.47	0.01591
A9P8D8	Peroxiredoxin- chloroplastic	5.04	0.00006	-	-
A0A067KWB6	Peroxiredoxin family protein	1.62	0.07771	3.40	0.01039
B0F824	Peroxygenase	5.48	0.00047	39.23	0.00023
I1MJC7	Phosphoglycerate cytosolic	4.04	0.00160	10.82	0.00067
A1BQH1	Phospho-glycerate partial	11.23	0.00001	18.09	0.00003
A0A067KYM7	Plasmodesmata callose-binding protein 3	0.39	0.00122	-	-
B9HYK9	Plus-3 domain-containing family protein	2.41	0.00114	-	-
Q9M549	Polyadenylate-binding protein 2	0.44	0.01491	-	-

A0A072UMK2	Polyubiquitin-a isoform x2	0.15	0.00000	1.83	0.14659
M5WVT8	Probable calcium-binding protein cml27	0.21	0.00390	0.34	0.00315
B3U2B1	Probable e3 ubiquitin-protein ligase log2	3.10	0.00306	0.41	0.01479
B9RIR5	Probable nadh dehydrogenase	-	-	2.81	0.03037
W9QKF5	Probable nadh dehydrogenase	0.46	0.18220	-	-
B6DQ61	Probable phospholipid hydroperoxide glutathione peroxidase	2.50	0.01323	-	-
B9RCA6	Probable phospholipid hydroperoxide glutathione peroxidase mitochondrial	0.80	0.00425	2.40	0.00035
V7CV47	Probable protein phosphatase 2c 55	11.25	0.00037	-	-
V7CD80	Probable serine protease eda2	0.64	0.07535	-	-
C6SVG8	Protease inhibitor seed storage ltp family protein	0.42	0.01119	-	-
W9R4F2	Proteasome subunit alpha type-2-a	7.96	0.02927	1.79	0.29097
B9IH97	Proteasome subunit alpha type-4	0.39	0.06878	-	-
A0A067KBK8	Proteasome subunit alpha type-7	1.69	0.00459	3.14	0.01495
I1JW44	Protein aspartic protease in guard cell 2-like	2.56	0.00042	2.01	0.00596
I1KP58	Protein dek	2.34	0.00984	-	-
Q9FRW1	Protein disulfide-isomerase	-	-	2.66	0.00200
A0A067K1B2	Protein efr3 homolog b-like isoform x2	2.52	0.00131	-	-
Q2PPJ2	Protein mother of ft and tf 1	3.94	0.00015	-	-
B9S2N7	Protein phosphatase	24.61	0.00001	-	-
B9T0Z8	Protein tic chloroplastic	0.14	0.00291	0.35	0.05516
B5AJV3	Ran-binding protein 1 homolog a-like	0.18	0.00281	0.43	0.13050
W9SHD4	Remorin family protein	0.22	0.02338	-	-
M5WTG1	Ribulose-phosphate 3- cytoplasmic isoform	5.86	0.07207	1.89	0.34807
E5GBB4	Seed maturation protein	-	-	3.83	0.02417
M5WJC3	Seed maturation protein pm41	0.03	0.00987	0.03	0.00342
B9SJ52	Serine carboxypeptidase s10 family protein	31.01	0.00036	-	-
I3SYA1	Serine carboxypeptidase-like 50	5.92	0.00025	-	-
W9QIF4	Short-chain type dehydrogenase reductase-like	-	-	2.11	0.00544
B9RMP5	Small heat shock chloroplastic-like	2.23	0.00154	-	-
H6TB40	Small heat shock chloroplastic-like isoform x2	5.76	0.02131	2.84	0.07108
A0A072UBI6	Small hydrophilic plant seed protein	-	-	2.13	0.01648
M5WJN3	Small ubiquitin-related modifier 1	0.17	0.01948	0.26	0.23354
O23759	Small ubiquitin-related modifier 1-like	0.32	0.00035	-	-
Q58A16	Stress responsive a b barrel domain	7.19	0.00005	-	-
B9TB09	Sulfate abc periplasmic sulfate-binding protein	-	-	4.12	0.00002
A0A072U725	Sulfite oxidase	8.09	0.00004	-	-
Q6QGY4	Superoxide dismutase	13.20	0.00012	-	-
A0A067KBA6	Syntaxin 73 family protein	0.09	0.00023	0.49	0.27029
K7M8K8	Thaumatin-like protein 1b	0.26	0.00821	-	-
A9PCJ7	Transcription factor btf3 homolog 4-like	25.42	0.00000	2.15	0.00007
Q9M5I8	Translationally-controlled tumor protein homolog	2.91	0.00000	2.57	0.00064
A0A072U2W1	Triosephosphate isomerase chloroplastic	6.60	0.00174	-	-
A0A067LKT3	Triosephosphate isomerase cytosolic	7.05	0.00842	14.10	0.15009
B9GJN0	Triosephosphate isomerase cytosolic	8.23	0.00026	11.29	0.00307

Q38IW8	Triosephosphate isomerase cytosolic	10.64	0.00037	11.03	0.00048
V7BC29	Tripeptidyl-peptidase 2-like isoform x1	0.22	0.00012	-	-
B9S382	Tubulin β-2 chain	-	-	19.71	0.00012
E5GB46	U2 small nuclear ribonucleoprotein a	10.22	0.00536	-	-
A0A067L974	Ubiquitin domain-containing protein dsk2a-like	0.13	0.00006	0.42	0.11805
B9H660	Ubiquitin domain-containing protein dsk2a-like	0.34	0.00007	0.38	0.00339
A6YTC7	Uncharacterized gpi-anchored protein at3g06035	0.53	0.10199	0.12	0.10064
A0A067JHV0	Uncharacterized gpi-anchored protein at5g19250-like	0.44	0.00329	-	-
Q19TV8	Utp--glucose-1-phosphate uridylyltransferase	0.29	0.00084	2.90	0.07416
Q9ZWI3	Vicilin gc72-a	3.49	0.00000	7.69	0.00000
Q39651	Vicilin-like antimicrobial peptides 2-2	6.54	0.00005	21.46	0.00001
F2YML9	Vicilin-like protein	-	-	8.47	0.00038
K7LJF5	Wat1-related protein at1g09380-like	45.88	0.00000	-	-

Red background indicates increased proteins, and green background indicates significant decreased protein (fold change ⩾2 or ⩽-2 and P value <0.05). "–"indicates not detected.

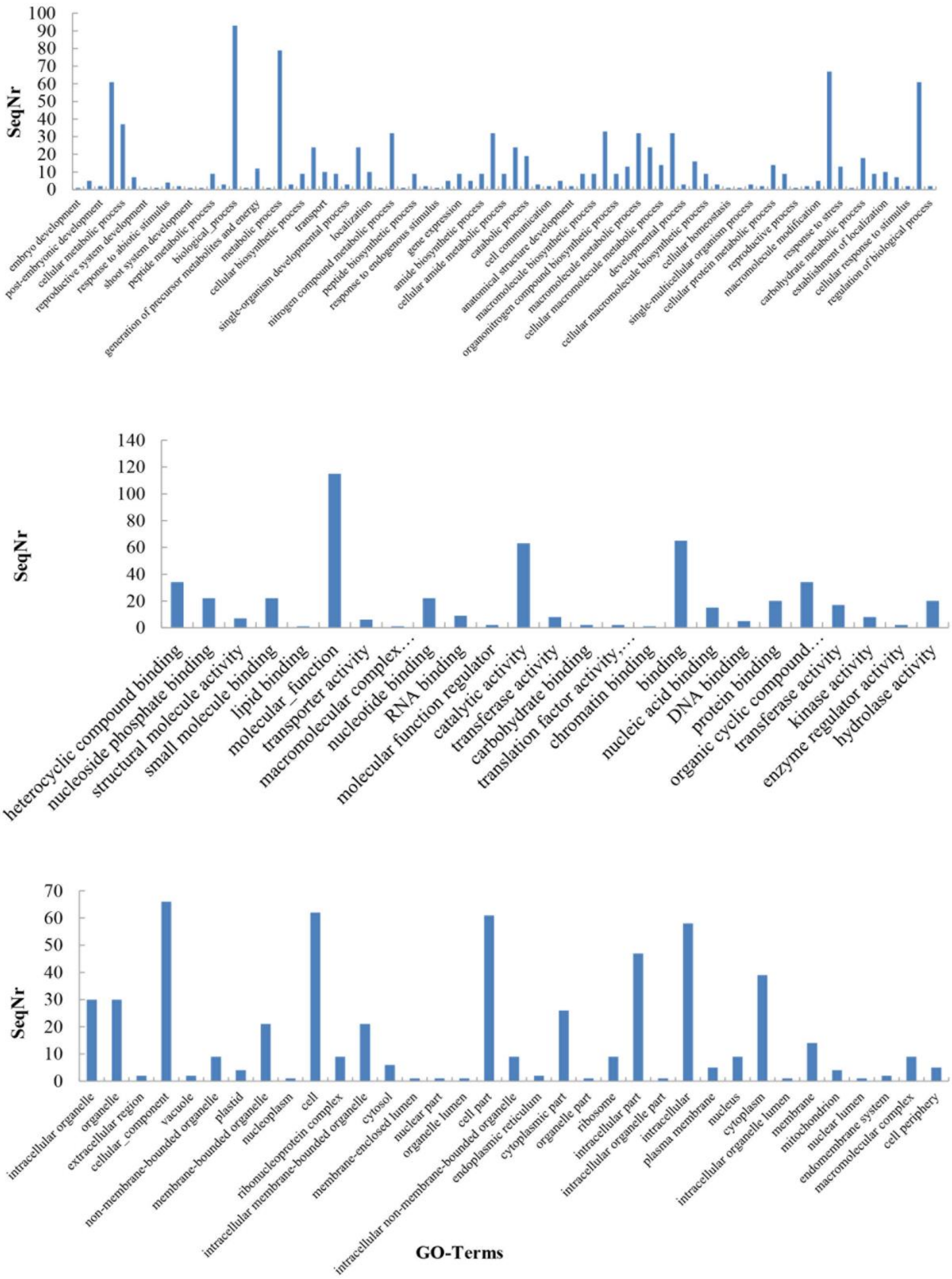

Fig. S1 Sequence distribution of differential expressed proteins involved in biological processes (A), molecule functions (B), and cellular components (C) after melatonin treatment under salt stress.

Glycolysis

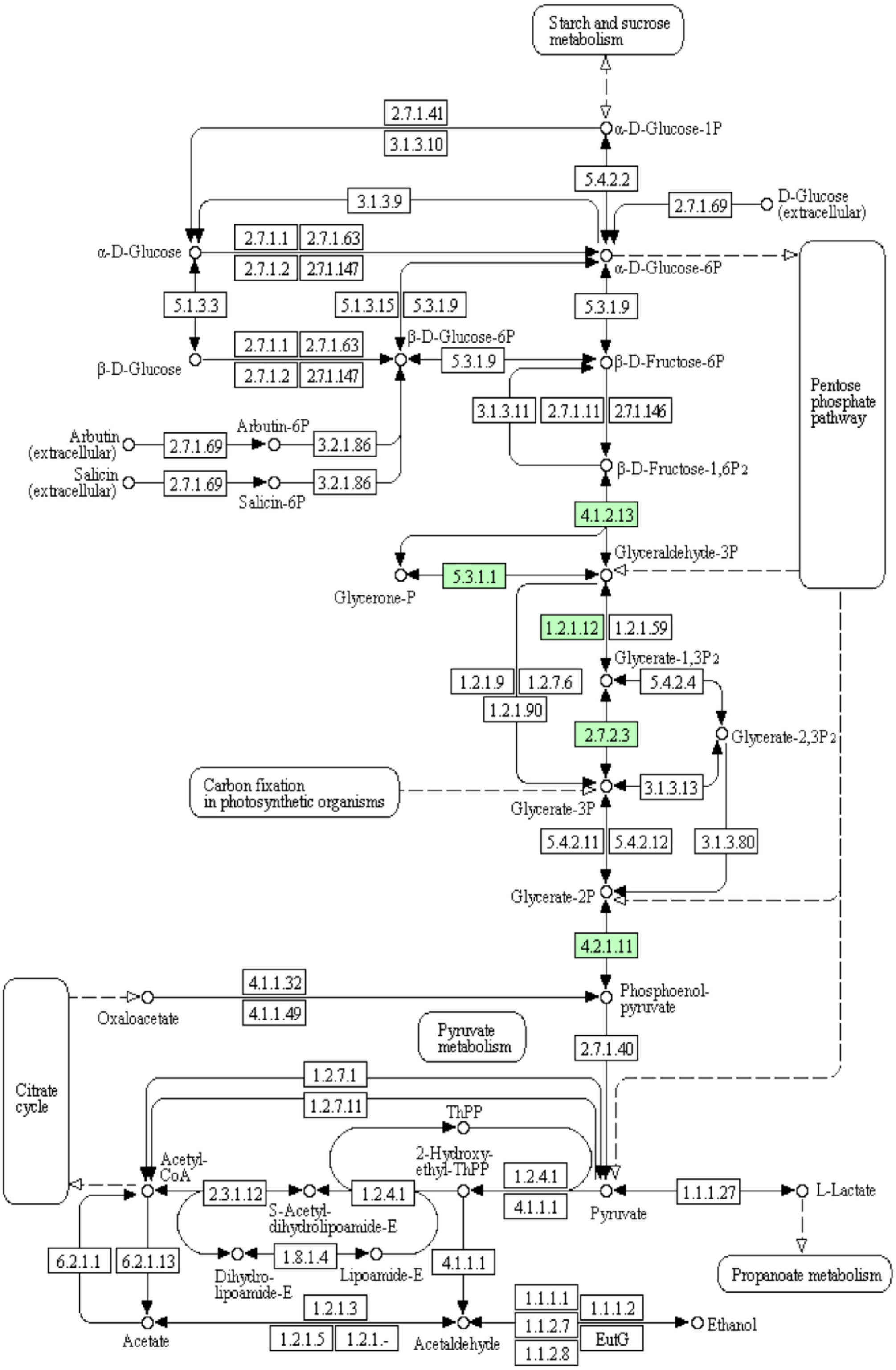

Fig.S2 Glycolysis pathway obtained from KEGG pathway analysis. The protein names in green color are differentially regulated by melatonin under salt stress during seed germination in cucumber.

Citrate cycle (TCA cycle)

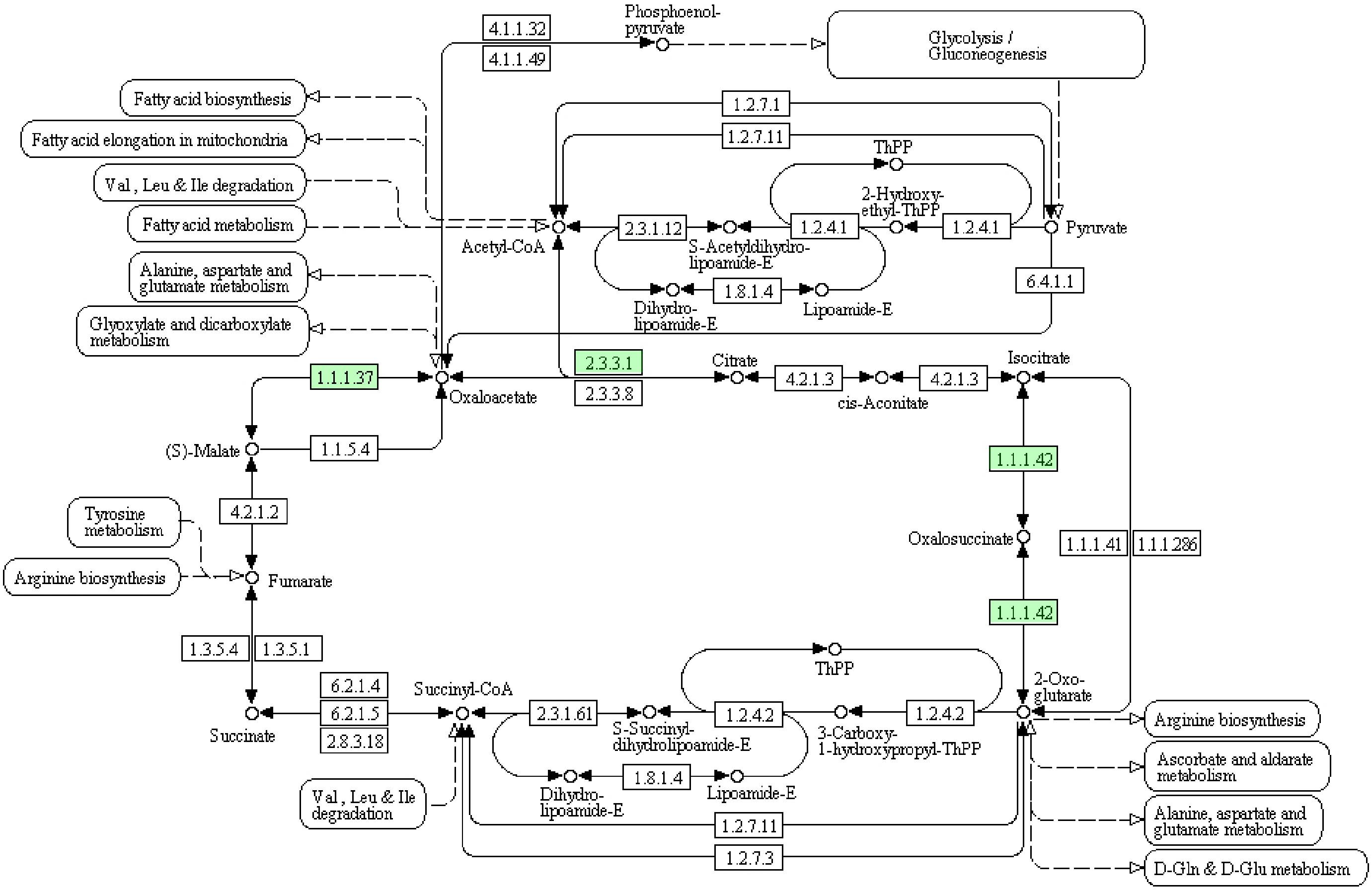

Fig.S3 TCA cycle pathway obtained from KEGG pathway analysis. The protein names in green color are differentially regulated by melatonin under salt stress during seed germination in cucumber.

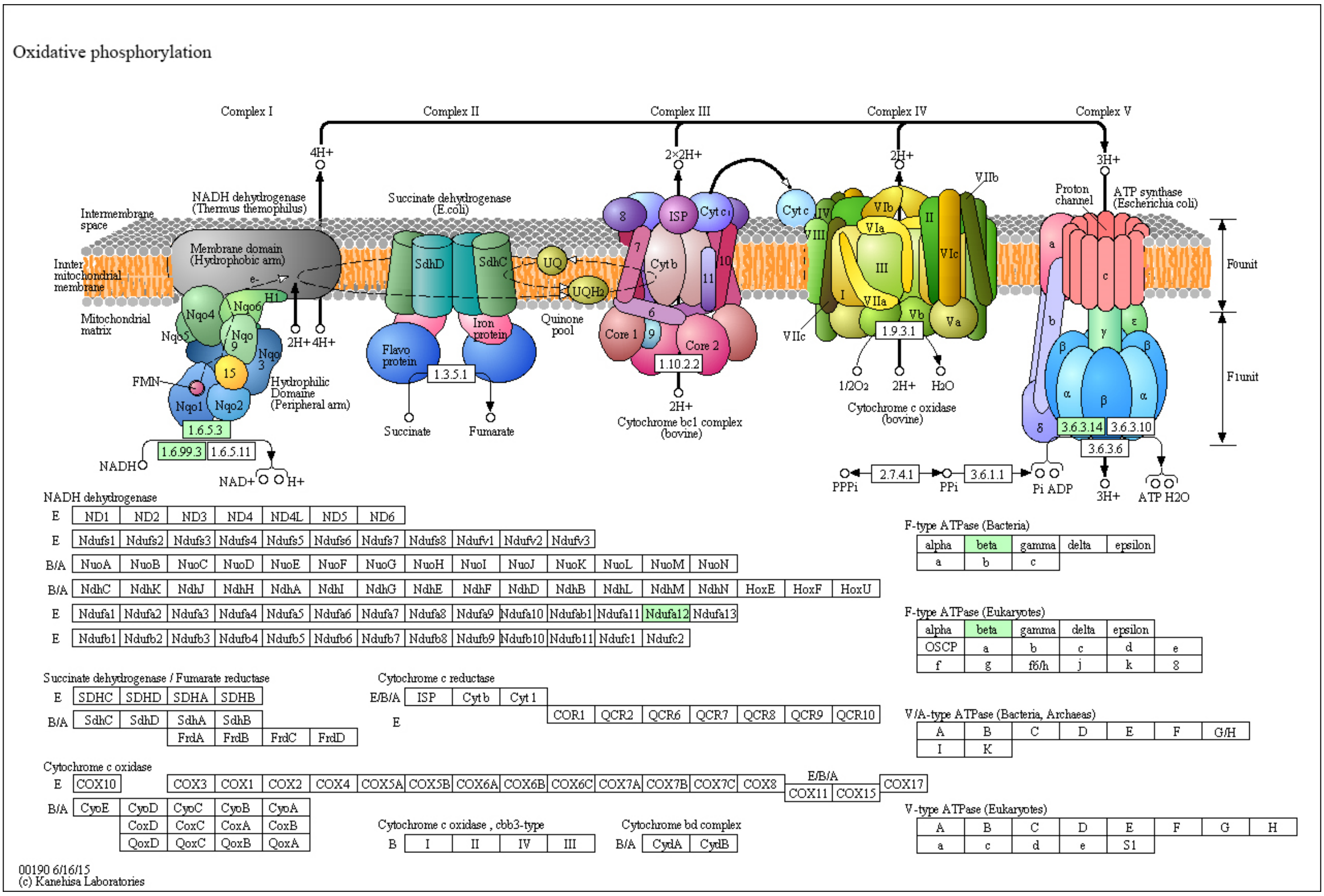

Fig.S4 Oxidative phosphorylation pathway obtained from KEGG pathway analysis. The protein names in green color are differentially regulated by melatonin under salt stress during seed germination in cucumber.
